# Benefit–risk profile of upadacitinib: exploratory post hoc analysis of phase 2b/3 studies in patients with moderately to severely active ulcerative colitis or Crohn’s disease

**DOI:** 10.1093/ecco-jcc/jjaf198

**Published:** 2025-11-20

**Authors:** Severine Vermeire, Jean-Frederic Colombel, Silvio Danese, Remo Panaccione, Laurent Peyrin-Biroulet, Kendall Beck, María Chaparro, Javier P Gisbert, Elena Dubcenco, Justin Klaff, Grace Naling, Sharanya Ford, Valencia Remple, Namita Joshi, Smitha Suravaram, Benjamin Duncan, Yibo Wang, Bettina Wick-Urban, Edward V Loftus

**Affiliations:** Department of Gastroenterology and Hepatology, University Hospitals of Leuven, Leuven 3000, Belgium; Henry D. Janowitz Division of Gastroenterology, Department of Medicine, Icahn School of Medicine at Mount Sinai, New York, NY 10029, United States; Gastroenterology and Endoscopy Unit, IRCCS Ospedale San Raffaele, Milan 20132, Italy; Inflammatory Bowel Disease Unit, Division of Gastroenterology and Hepatology, University of Calgary, Calgary, Alberta AB T2N 1N4, Canada; Department of Gastroenterology, CHRU Nancy, INSERM NGERE, Université de Lorraine, Vandœuvre-lès-Nancy F-54500, France; Division of Gastroenterology and Hepatology, McGill University Health Centre, Montreal, Quebec, QC H4A 0B1, Canada; Division of Gastroenterology and Hepatology, Department of Medicine, University of California, San Francisco, CA 94143, United States; Gastroenterology Unit, Hospital Universitario de La Princesa, Instituto de Investigación Sanitaria Princesa (IIS-Princesa), Universidad Autónoma de Madrid (UAM), Centro de Investigación Biomédica en Red de Enfermedades Hepáticas y Digestivas (CIBEREHD), Madrid 28006, Spain; Gastroenterology Unit, Hospital Universitario de La Princesa, Instituto de Investigación Sanitaria Princesa (IIS-Princesa), Universidad Autónoma de Madrid (UAM), Centro de Investigación Biomédica en Red de Enfermedades Hepáticas y Digestivas (CIBEREHD), Madrid 28006, Spain; AbbVie Inc., North Chicago, IL 60044, United States; AbbVie Inc., North Chicago, IL 60044, United States; AbbVie Inc., North Chicago, IL 60044, United States; AbbVie Inc., North Chicago, IL 60044, United States; AbbVie Inc., North Chicago, IL 60044, United States; AbbVie Inc., North Chicago, IL 60044, United States; AbbVie Inc., North Chicago, IL 60044, United States; AbbVie Inc., North Chicago, IL 60044, United States; AbbVie Inc., North Chicago, IL 60044, United States; AbbVie Deutschland GmbH & Co. KG, Ludwigshafen 67061, Germany; Division of Gastroenterology and Hepatology, Mayo Clinic College of Medicine and Science, Rochester, MN 55905, United States

**Keywords:** Crohn’s disease, ulcerative colitis, upadacitinib

## Abstract

**Background and Aims:**

Upadacitinib (UPA)—an oral, reversible selective Janus kinase inhibitor—has a favorable benefit–risk profile for patients with Crohn’s disease (CD) and ulcerative colitis (UC). We evaluated the benefit–risk of UPA in select subgroups with CD or UC.

**Methods:**

Patients were randomized to UPA 45 mg (UPA45) once daily (QD) or placebo (PBO) induction for 12 (CD: U-EXCEED, U-EXCEL) or 8 weeks (UC: U-ACHIEVE, U-ACCOMPLISH). Clinical responders were re-randomized to QD UPA 15 mg (UPA15), UPA 30 mg (UPA30), or PBO for 52-week maintenance (CD: U-ENDURE; UC: U-ACHIEVE). This exploratory post hoc analysis assessed efficacy and safety outcomes (adverse events of special interest [AESIs]: serious infections, major adverse cardiovascular [CV] events, malignancies, and venous thromboembolic events) by CV risk, prior treatment failure, and age.

**Results:**

This analysis included 1021 patients with CD and 1097 with UC during induction, and 673 with CD and 746 with UC during maintenance. Improved efficacy outcomes comparable to the overall study populations were observed with UPA versus PBO across subgroups. Patients receiving UPA30 generally showed numerically higher rates of improvements versus UPA15. AESI rates were generally comparable between UPA and PBO across subgroups except for numerically higher rates of herpes zoster and serious infections in CD with UPA.

**Conclusions:**

UPA resulted in consistent benefit versus placebo across CV risk, prior treatment failure, and age subgroups. No treatment differences were seen in AESIs across subgroups except herpes zoster and serious infections, reinforcing the favorable benefit–risk profile for UPA in CD and UC seen in the overall study populations.

**Clinical trial numbers:**

NCT02819635, NCT03653026, NCT03345836, NCT03345849, NCT03345823

## 1. Introduction

Inflammatory bowel disease (IBD) is a chronic, relapsing-remitting inflammatory disease that includes Crohn’s disease (CD) and ulcerative colitis (UC).^1^ Common symptoms of IBD include diarrhea, abdominal pain, nausea, and vomiting.^1^ CD can impact the entire digestive tract and is characterized by the presence of transmural inflammatory lesions, whereas UC is limited to the colon and rectum and is characterized by mucosal inflammation and ulceration.^2^^,^^3^

Janus kinase (JAK) inhibitors (JAKis) have emerged as targeted therapeutics for IBD.^4^^,^^5^ By inhibiting various JAK isoforms, these agents partially interrupt the signaling pathway of multiple pro-inflammatory cytokines, thereby reducing the inflammatory and immune responses seen in IBD.^6^ Upadacitinib is an oral, reversible, selective inhibitor of JAK1 with demonstrated efficacy in inducing and maintaining clinical and endoscopic remission in patients with moderately to severely active CD (U-EXCEED, U-EXCEL, and U-ENDURE)^7^ and in patients with moderately to severely active UC (U-ACCOMPLISH and U-ACHIEVE), with an acceptable safety profile.^8^^,^^9^

Safety considerations arose during JAKi development in rheumatoid arthritis (RA). The Oral RA Trial (ORAL) Surveillance study of tofacitinib was conducted in an at risk population aged ≥50 years with RA who also had at least one cardiovascular (CV) risk factor (current cigarette smoker, hypertension, high-density lipoprotein cholesterol level of <40 mg/dL, diabetes mellitus, family history of premature coronary heart disease, extra-articular RA, or history of coronary artery disease).^10^ The results of the trial indicated potential safety concerns with JAKis, with major adverse CV events (MACE) and malignancies occurring more often with tofacitinib treatment than with a tumor necrosis factor (TNF) inhibitor, and secondary analyses showing higher rates of adverse events of special interest (AESIs) including herpes zoster and venous thromboembolic events (VTEs) among patients treated with tofacitinib compared with a TNF inhibitor.^10^ Given differences in disease pathophysiology and the fact that IBD typically affects a younger population, the risks demonstrated with tofacitinib should not be directly extrapolated to upadacitinib and the IBD population.

To aid in treatment decision-making, this post hoc analysis aimed to evaluate the benefits and risks of upadacitinib compared to placebo in patients from the CD and UC phase 2b/3 clinical trials in different subgroups by key demographics including those with CV risk factors, prior treatment failure, and age.

## 2. Methods

The study details for the 2 induction CD studies, 1 maintenance CD study, 2 induction UC studies, and 1 maintenance UC study were previously described in detail^7–9^ and are summarized below.

### Study design

Patients eligible for the CD induction studies were aged 18-75 years and had moderately to severely active CD for ≥3 months, defined as an average daily very soft or liquid stool frequency (SF) score ≥4 and/or average daily abdominal pain score (APS) ≥2, and a centrally reviewed Simple Endoscopic Score for CD (SES-CD) of ≥6, or ≥4 for isolated ileal disease, excluding the narrowing component. Patients who achieved clinical response to 12 weeks of upadacitinib 45 mg (UPA45) once daily (QD) as induction therapy in U-EXCEL (NCT03345849) or U-EXCEED (NCT03345836) entered the 52-week U-ENDURE (NCT03345823) maintenance study.

Patients eligible for the UC induction studies U-ACCOMPLISH (NCT03653026) and U-ACHIEVE (NCT02819635) were aged 16-75 years with a confirmed diagnosis of UC for ≥90 days before study entry; active disease (as defined by an Adapted Mayo Score of 5-9 and a centrally assessed Mayo endoscopic subscore of 2 or 3); and an inadequate response or intolerance to ≥1 oral aminosalicylate, corticosteroid, immunosuppressant, or biological therapy. Clinical responders to 8 weeks of UPA45 in the phase 2b induction study, or the phase 3 induction study, were randomized to receive double-blind treatment with upadacitinib 15 mg (UPA15) QD, upadacitinib 30 mg (UPA30) QD, or placebo for 52 weeks in the phase 3 maintenance study U-ACHIEVE.

### Outcomes

This post hoc analysis evaluated the efficacy and safety of upadacitinib by subgroups (CV risk factors, prior treatment failure, and age) and in the overall population. To assess the benefits and risks of upadacitinib, efficacy and safety outcomes in the trials were assessed at week 12 (CD) and week 8 (UC) of the induction period and week 52 (CD and UC) of the maintenance period in the subgroups and in the overall population. Subgroups were assessed by CV risk at baseline (low or high risk), by prior treatment failure (patients with or without inadequate response, loss of response, or intolerance to a biologic inadequate response [bio-IR] or non-bio-IR, respectively]), by previous inadequate response to anti-tumor necrosis factor (TNF-IR) therapy (within the bio-IR population), and by age (<50, 50-64, or ≥65 years). CV risk factors used to classify patients as high risk included age ≥65 years, Black or African American race, body mass index (BMI) ≥30 kg/m², current or former (within 15 years) tobacco smoker, excess alcohol use (>4 drinks/day), a history of CV disease or other relevant cardiac problems, diabetes mellitus, hypertension, VTE/thrombosis of limbs or major organs, chronic kidney disease, baseline or prior antihypertensive use, decreased HDL cholesterol level (<40 mg/dL), or elevated blood pressure. Patients with ≥1 CV risk factor were designated as being at a higher risk for a CV event; all other patients were designated as low CV risk.

For CD, the outcomes assessed were the CD Activity Index (CDAI) clinical remission, SF/APS clinical remission, ­maintenance of clinical remission (per CDAI or SF/APS), glucocorticoid-free clinical remission (per CDAI or SF/APS), clinical response by the CDAI score, endoscopic response, endoscopic remission, deep remission (clinical remission and endoscopic remission), change from baseline in responses on the IBD Questionnaire (IBDQ), and change from baseline in fatigue (Functional Assessment of Chronic Illness Therapy-Fatigue [FACIT-F]).

For UC, the selected end points were clinical remission per the Adapted Mayo Score; maintenance of clinical response per the Adapted Mayo Score at week 52 among patients who achieved clinical response per the Adapted Mayo Score at the end of the induction period; maintenance of clinical remission per the Adapted Mayo Score; corticosteroid-free clinical remission per the Adapted Mayo Score; endoscopic improvement; endoscopic remission; histological endoscopic mucosal improvement; mucosal healing; no abdominal pain; no bowel urgency; change from baseline in the FACIT-F score; and change from baseline in responses on the IBDQ. Efficacy end points for both CD and UC are defined in the Supplementary Appendix.

Safety was assessed according to treatment-emergent adverse events (TEAEs) reported through the induction and maintenance periods among patients who received ≥1 dose of upadacitinib or placebo. TEAEs were coded using the Medical Dictionary for Regulatory Activities (version 23.0 for UC; version 24.0 for CD). The severity of adverse events (AEs) and laboratory abnormalities were graded using the Common Terminology Criteria for AEs (version 4.03). The AESIs, which were evaluated as part of this benefit–risk assessment for upadacitinib, were serious infections, herpes zoster, malignancy excluding nonmelanoma skin cancer (NMSC), NMSC, adjudicated gastrointestinal perforations, adjudicated MACE (defined as CV death, nonfatal myocardial infarction, and nonfatal stroke), and adjudicated VTE (defined as deep vein thrombosis and pulmonary embolism [fatal and nonfatal]).

### Statistical analysis

Descriptions of the statistical analyses for the studies included here were previously reported.^7^^,^^8^ Efficacy and safety analyses were conducted in the intent-to-treat populations, defined as the UPA45 12-week (CD) or 8-week (UC) induction responders who were randomized per protocol for the 52-week maintenance treatment period and received ≥1 dose of study drug (placebo, UPA15, or UPA30). Rate differences (with 95% confidence interval [CI]) evaluating the benefit of upadacitinib versus placebo were calculated using the normal approximation to the binomial distribution, utilizing nonresponder imputation incorporating multiple imputation to handle missing data due to COVID-19 or nonresponder imputation only if there were no missing data due to COVID-19.

Change from baseline end points for induction were assessed using least square means, which were calculated with mixed-effects models for repeated measurements, incorporating baseline value, treatment, visit, treatment-by-visit interaction, and stratification factors in the model using an unstructured covariance matrix. For change from baseline end points for maintenance, least squares means were calculated using mixed-effects models for repeated measurements. The baseline induction value, week 0 value, stratification factors, treatment, visit, and treatment-by-visit interaction were included using an unstructured covariance matrix. If the model could not converge, the autoregressive (1) or covariance structure matrix was used. Study-sized adjusted (SSA) risk differences in binomial proportions were computed with corresponding 95% Cls from the modified stratified Wald method (adapted to SSA weights) for all UC analyses and for induction analyses for CD.^11^

For CD maintenance analyses, unadjusted risk differences in binomial proportions were computed with corresponding 95% CIs derived from Wald-based asymptotic methods that were based on the normal approximation to the binomial distribution. However, when there were ≤3 total patients with the event across the two respective treatment groups, exact CIs were derived by inverting two separate one-sided exact tests based on the unstandardized risk difference.^12^

Risk differences and their corresponding CIs were computed for UPA45 versus placebo in the induction analyses. In the maintenance analyses, these calculations were made for UPA30 versus placebo and UPA15 versus placebo. Calculations were done by indication, for the overall population, and for the following subgroups as specified: CV risk (low, high), previous treatment status (previous treatment failure [bio-IR, non-bio-IR; UC and CD—maintenance], prior biologic or TNF response [bio-IR, non-bio-IR, TNF-IR within bio-IR; UC and CD—induction], prior inadequate response to anti-TNF [TNF-IR within bio-IR; UC and CD—maintenance]), and age group (ages <50, 50–64, and ≥65).

### Ethical requirements

The trials were conducted in accordance with the International Council for Harmonization guidelines, applicable regulations, and the Declaration of Helsinki. An independent ethics committee or institutional review board at each trial site approved the protocol. The study protocol, informed consent forms, and recruitment materials were approved by the relevant ethics committees or institutional review boards of each country before enrollment. All patients provided written informed consent.

## 3. Results

### Patients

Baseline demographics and clinical characteristics of patients were generally well-balanced across indications and treatment groups (Table 1 and Table S1) and have been previously described.^7^^,^^8^

**Table 1.** Patient demographics and clinical characteristics at maintenance baseline.

Parameter, *n* (%)	Ulcerative colitis				Crohn’s Disease		
	Placebo (*N* = 245)	UPA 15 mg QD (*N* = 250)	UPA 30 mg QD (*N* = 251)	Placebo (*N* = 223)		UPA 15 mg QD (*N* = 221)	UPA 30 mg QD (*N* = 229)
**Female**	111 (45.3)	91 (36.4)	97 (38.6)	111 (49.8)		87 (39.4)	98 (42.8)
**Age, years, mean (SD)**	42.7 (14.5)	41.9 (14.2)	42.8 (14.5)	38.5 (13.1)		37.5 (13.4)	37.0 (13.0)
**Age group, years**							
** <50**	156 (63.7)	175 (70.0)	172 (68.5)	171 (76.7)		178 (80.5)	185 (80.8)
** 50–64**	68 (27.8)	52 (20.8)	58 (23.1)	44 (19.7)		36 (16.3)	36 (15.7)
** ≥65**	21 (8.6)	23 (9.2)	21 (8.4)	8 (3.6)		7 (3.2)	8 (3.5)
**Race**							
** White**	157 (64.1)	163 (65.2)	166 (66.1)	170 (76.2)		165 (74.7)	160 (69.9)
** Black or African American**	7 (2.9)	9 (3.6)	6 (2.4)	11 (4.9)		8 (3.6)	10 (4.4)
** Asian**	71 (29.0)	77 (30.8)	76 (30.3)	40 (17.9)		46 (20.8)	57 (24.9)
**American Indian or Alaska Native**	1 (0.4)	0	0	0		0	0
**Native Hawaiian or Other Pacific Islander**	1 (0.4)	0	1 (0.4)	0		0	0
** Multiple**	8 (3.3)	1 (0.4)	2 (0.8)	2 (0.9)		2 (0.9)	2 (0.9)
**BMI, kg/m^2^, mean (SD)**	25.0 (5.5)	24.9 (5.7)	25.3 (5.9)	24.7 (6.4)		24.2 (5.9)	24.3 (6.3)
**BMI**							
** <30**	203 (82.9)	207 (82.8)	206 (82.1)	182 (81.6)		177 (80.1)	185 (80.8)
** ≥30**	42 (17.1)	43 (17.2)	43 (17.1)	41 (18.4)		44 (19.9)	44 (19.2)
** Missing**	0	0	2 (0.8)	0		0	0
**Years since diagnosis, mean (SD)**	8.7 (8.1)	8.3 (7.3)	8.0 (6.9)	10.1 (8.9)		10.5 (8.6)	9.5 (8.6)
**Adapted Mayo Score, mean (SD)**	7.0 (1.2)	6.9 (1.2)	7.0 (1.3)	–		–	–
**CDAI, mean (SD)**	–	–	–	305.6 (82.5)		299.7 (88.8)	305.0 (79.9)
**SES-CD, mean (SD)**	–	–	–	15.0 (7.6)		15.6 (7.6)	14.9 (7.8)
**SES-CD**							
** <15**	–	–	–	123 (55.2)		112 (50.7)	131 (57.2)
** ≥15**				100 (44.8)		109 (49.3)	98 (42.8)
**CV risk factors**							
** Any CV risk factor**	155 (63.3)	137 (54.8)	154 (61.4)	145 (65.0)		142 (64.3)	138 (60.3)
** Aged ≥65 years**	21 (8.6)	23 (9.2)	21 (8.4)	8 (3.6)		7 (3.2)	8 (3.5)
** Black or African American**	7 (2.9)	9 (3.6)	6 (2.4)	11 (4.9)		8 (3.6)	10 (4.4)
** BMI ≥30 kg/m^2^**	42 (17.1)	43 (17.2)	43 (17.1)	41 (18.4)		44 (19.9)	44 (19.2)
** Current tobacco smoker**	25 (10.2)	19 (7.6)	24 (9.6)	45 (20.2)		38 (17.2)	43 (18.8)
**Tobacco smoker within the past 15 years**	45 (18.4)	52 (20.8)	51 (20.3)	25 (11.2)		38 (17.2)	28 (12.2)
**Current alcohol consumption ≥4 drinks/day**	6 (2.4)	5 (2.0)	8 (3.2)	2 (0.9)		2 (0.9)	2 (0.9)
**History of CV disease**	18 (7.3)	12 (4.8)	14 (5.6)	9 (4.0)		6 (2.7)	8 (3.5)
**History of diabetes mellitus**	12 (4.9)	15 (6.0)	16 (6.4)	9 (4.0)		4 (1.8)	7 (3.1)
**History of hypertension**	46 (18.8)	28 (11.2)	40 (15.9)	34 (15.2)		20 (9.0)	31 (13.5)
**History of VTE**	4 (1.6)	3 (1.2)	5 (2.0)	4 (1.8)		4 (1.8)	2 (0.9)
**History of chronic ­kidney disease**	0	3 (1.2)	1 (0.4)	2 (0.9)		3 (1.4)	0
**Prior antihypertensive use**	2 (0.8)	0	1 (0.4)	1 (0.4)		0	0
**Decreased HDL cholesterol (<40 mg/dL)**	30 (12.2)	30 (12.0)	42 (16.7)	36 (16.1)		55 (24.9)	47 (20.5)
**Elevated blood pressure**	19 (7.8)	13 (5.2)	16 (6.4)	18 (8.1)		8 (3.6)	10 (4.4)
**Previous biologic history**							
** Without failure**	112 (45.7)	124 (49.6)	128 (51.0)	53 (23.8)		57 (25.8)	59 (25.8)
** With failure**	133 (54.3)	126 (50.4)	123 (49.0)	170 (76.2)		164 (74.2)	170 (74.2)
** >1 biologic**	85 (34.7)	77 (30.8)	81 (32.3)	106 (47.5)		101 (45.7)	113 (49.3)
**≥1 Anti-TNF failure**	43 (17.6)	35 (14.0)	39 (15.5)	63 (28.3)		67 (30.3)	72 (31.4)
**Herpes zoster vaccination by age group, years**	7 (2.9)	5 (2.0)	12 (4.8)	12 (5.4)		16 (7.2)	17 (7.4)
** <50**	1 (0.4)	2 (0.8)	8 (3.2)	7 (3.1)		10 (4.5)	11 (4.8)
** 50–64**	3 (1.2)	2 (0.8)	1 (0.4)	3 (1.3)		2 (0.9)	5 (2.2)
**≥65**	3 (1.2)	1 (0.4)	3 (1.2)	2 (0.9)		4 (1.8)	1 (0.4)

BMI, body mass index; CDAI, Crohn’s Disease Activity Index; CV, cardiovascular; QD, once daily; SD, standard deviation; SES-CD, Simple Endoscopic Score for Crohn’s Disease; TNF, tumor necrosis factor; UPA, upadacitinib; VTE, venous thromboembolism.

A total of 1021 patients with CD were randomly assigned to receive UPA45 (*N* = 674) or placebo (*N* = 347) in the U-EXCEL and U-EXCEED induction studies and were included in this analysis. A total of 673 patients with clinical response to UPA45 induction therapy received placebo (*N* = 223), UPA15 (*N* = 221), or UPA30 (*N* = 229) in the 52-week U-ENDURE maintenance study and were included in this analysis. A total of 1097 patients with UC were randomly assigned to receive UPA45 (*N* = 719) or placebo (*N* = 378) in the U-ACHIEVE and U-ACCOMPLISH induction studies and were included in this analysis. A total of 746 patients with clinical response to UPA45 induction therapy received placebo (*N* = 245), UPA15 (*N* = 250), or UPA30 (*N* = 251) in the 52-week maintenance study U-ACHIEVE and were included in this analysis.

### Benefit–risk profile of upadacitinib in CD and UC across subgroups

#### CV risk (low vs high)

Across CV risk subgroups, patients with CD receiving UPA45 during the induction period showed greater clinical benefit versus those in the placebo group. Patients receiving upadacitinib had greater rates of clinical remission per CDAI, clinical remission per SF/APS, endoscopic response, and endoscopic remission versus placebo (Figure S1). The rates of AESI with UPA45 treatment and placebo during the induction period were low and comparable across CV risk groups (response rate difference [95% CI]), except for higher rates of herpes zoster with UPA45 (low risk, 3.3 [0.4, 6.2]; high risk, 1.6 [0.1, 3.2]).

**Figure 1.**
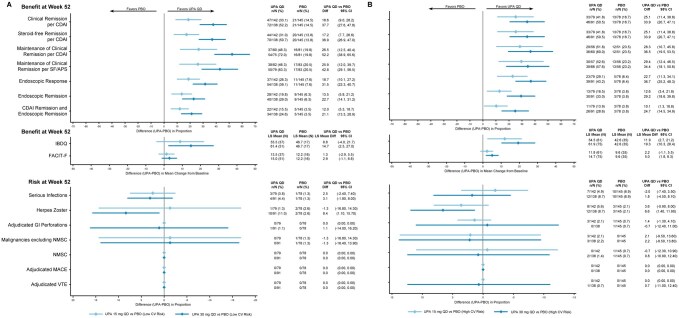
Benefit–risk during maintenance by CV risk factors in patients with CD. (A) Low CV risk factors. (B) High CV risk factors. APS, abdominal pain score; CD, Crohn’s disease; CDAI, Crohn’s Disease Activity Index; CV, cardiovascular; FACIT-F, Functional Assessment of Chronic Illness Therapy-Fatigue; GI, gastrointestinal; IBDQ, Inflammatory Bowel Disease Questionnaire; LS, least squares; MACE, major adverse cardiovascular event; NMSC, nonmelanoma skin cancer; PBO, placebo; QD, once daily; SF, stool frequency; UPA, upadacitinib; VTE, venous thromboembolic event. CV risk factors in this analysis used to classify patients as high risk included age ≥65 years, Black or African American race, BMI ≥30 kg/m², current or former (within 15 years) tobacco smoker, excess alcohol use (>4 drinks/day), a history of CV disease or other relevant cardiac problems, diabetes mellitus, hypertension, VTE/thrombosis of limbs or major organs, chronic kidney disease, baseline or prior hypertensive use, decreased HDL cholesterol (<40 mg/dL), or elevated blood pressure. A patient meeting at least one of the previously identified CV risk factors are designated as high CV risk, whereas all other patients are designated as low CV risk.

During the maintenance period, efficacy outcomes favored UPA15 and UPA30 versus placebo (response rate difference) across CV risk subgroups (*CDAI*, low risk: UPA15, 18.6; UPA30: 37.7; high risk: UPA15, 25.1; UPA30, 33.9; *SF/APS,* low risk: UPA15, 15.8; UPA30, 32.6; high risk: UPA15, 23.8; UPA30, 36.1; *endoscopic response*, low risk: UPA15, 18.7; UPA30, 31.5; UPA15, 22.7; UPA30, 36.7; *endoscopic remission*, low risk: UPA15, 13.5; UPA30, 22.7; high risk: UPA15, 12.6; UPA30, 29.2; Figure 1).

The AESI rates during the maintenance period were low and comparable for the upadacitinib and placebo groups, except for herpes zoster and a numerically higher rate of serious infections in the UPA30 group. The risks were generally comparable for patients with low or high CV risk, except for herpes zoster, where the risk was comparable for UPA15 and placebo and greater for UPA30 vs placebo (*herpes zoster,* low risk: UPA15, −1.3 [−16.8, 14.3]; UPA30, 8.4 [1.1, 15.7]; high risk: UPA15, 3.6 [−0.9, 8.0]; UPA30, 6.6 [1.4, 11.9]; *serious infections,* low risk: UPA15, 2.5 [−2.4, 7.4]; UPA30, 3.1 [−1.8, 8.0]; high risk: UPA15, −2.0 [−7.4, 3.5]; UPA30, 1.8 [−4.5, 8.1]).

For UC, efficacy outcomes for patients with low or high CV risk factors were favorable for UPA45 versus placebo after 8 weeks of treatment across the evaluated end points during the induction period (Figure S2). The rates of AESI were low and comparable between UPA45 and placebo.

**Figure 2.**
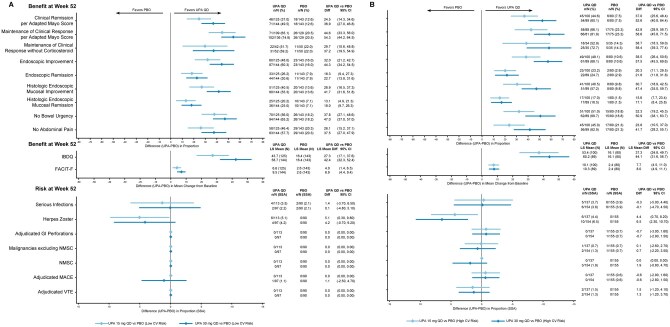
Benefit–risk during maintenance by CV risk factors in patients with UC. (A) Low CV risk factors. (B) High CV risk factors. CV, cardiovascular; FACIT-F, Functional Assessment of Chronic Illness Therapy-Fatigue; GI, gastrointestinal; IBDQ, Inflammatory Bowel Disease Questionnaire; LS, least squares; MACE, major adverse cardiovascular event; NMSC, nonmelanoma skin cancer; PBO, placebo; QD, once daily; SSA, study-size adjusted; UC, ulcerative colitis; UPA, upadacitinib; VTE, venous thromboembolic event. CV risk factors in this analysis used to classify patients as high risk included age ≥65 years, Black or African American race, BMI ≥30 kg/m², current or former (within 15 years) tobacco smoker, excess alcohol use (>4 drinks/day), a history of CV disease or other relevant cardiac problems, diabetes mellitus, hypertension, VTE/thrombosis of limbs or major organs, chronic kidney disease, baseline or prior hypertensive use, decreased HDL cholesterol (<40 mg/dL), or elevated blood pressure. A patient meeting at least one of the previously identified CV risk factors are designated as high CV risk, whereas all other patients are designated as low CV risk.

During the maintenance period, improved clinical outcomes were observed with both upadacitinib doses compared with placebo across CV risk subgroups, with the greatest improvement generally observed with UPA30 vs placebo (*clinical remission per adapted Mayo Score,* low risk: UPA15, 24.5; UPA30, 36.9; high risk: UPA15, 37.0; UPA30, 52.6; *maintenance of clinical response per adapted Mayo Score*, low risk: UPA15, 44.6; UPA30, 54.3; high risk: UPA15, 42.8; UPA30, 58.6; *endoscopic improvement,* low risk: UPA15, 32.0; UPA30, 44.3; high risk: UPA15, 38.5; UPA30, 57.5; *endoscopic remission,* low risk: UPA15, 18.3; UPA30, 22.7; high risk: UPA15, 20.3; UPA30, 21.8; Figure 2).

The AESI rates during the maintenance period were low with similar rates for the upadacitinib and placebo groups, except for higher rates of herpes zoster with both upadacitinib doses versus placebo for patients with low or high CV risk (low risk: UPA15, 5.1 [0.3, 9.8]; UPA30, 4.2 [−0.7, 9.2]; high risk: UPA15, 4.4 [0.7, 8.2]; UPA30, 6.5 [2.3, 10.7]).

#### Prior treatment failure

Among patients with CD, the differences between UPA45 and placebo for the efficacy outcomes during the induction period were numerically higher for the bio-IR and TNF-IR subgroups compared with the non-bio-IR subgroup (Figure S3).

**Figure 3.**
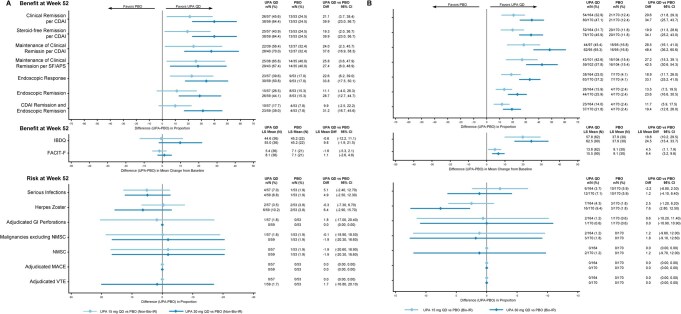
Benefit–risk during maintenance by previous treatment failure in patients with CD. Patients (A) with or (B) without ­inadequate response, loss of response, or intolerance to a biologic (non-bio-IR, or bio-IR, respectively). APS, abdominal pain score; CD, Crohn’s disease; CDAI, Crohn’s Disease Activity Index; FACIT-F, Functional Assessment of Chronic Illness Therapy-Fatigue; GI, gastrointestinal; IBDQ, Inflammatory Bowel Disease Questionnaire; IR, inadequate response; LS, least squares; MACE, major adverse cardiovascular event; NMSC, ­nonmelanoma skin cancer; PBO, placebo; QD, once daily; SF, stool frequency; UPA, upadacitinib; VTE, venous thromboembolic event.

During the maintenance period, the rate differences were generally consistent for efficacy outcomes (response rate difference) across the non-bio-IR, bio-IR, and TNF-IR subgroups (*CDAI*, bio-IR: UPA15, 20.6; UPA30, 34.7; non-bio-IR: UPA15, 21.1; UPA30, 39.9; *SF/APS*, bio-IR: UPA15, 19.9; UPA30, 33.5; non-bio-IR: UPA15, 13.5; UPA30, 34.0; *endoscopic response*, bio-IR: UPA15, 18.9; UPA30: 33.1; non-bio-IR: UPA15, 22.6; UPA30, 33.8; *endoscopic remission*, bio-IR: UPA15, 13.5; UPA30: 23.6; non-bio-IR: UPA15, 11.1; UPA30, 28.7; Figure 3 and Figure S4). Numerically greater improvements were observed across efficacy outcomes with UPA30 compared with UPA15.

**Figure 4.**
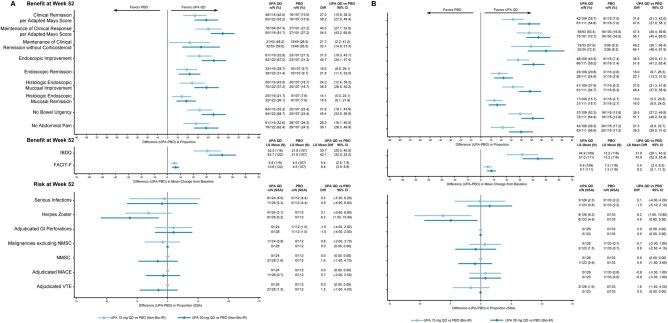
Benefit–risk during maintenance by previous treatment failure in patients with UC. Patients (A) with or (B) without inadequate response, loss of response, or intolerance to a biologic (non-bio-IR, or bio-IR, respectively). FACIT-F, Functional Assessment of Chronic Illness Therapy-Fatigue; GI, gastrointestinal; IBDQ, Inflammatory Bowel Disease Questionnaire; IR, inadequate responder; LS, least squares; MACE, major adverse cardiovascular event; NMSC, nonmelanoma skin cancer; PBO, placebo; QD, once daily; SSA, study-size adjusted; UC, ulcerative colitis; UPA, upadacitinib; VTE, venous thromboembolic event.

Higher rates of herpes zoster (response rate difference [95% CI]) were observed with upadacitinib compared with placebo during the maintenance period, regardless of subgroup (bio-IR: UPA15, 2.5 [−1.2, 6.2]; UPA30, 7.6 [2.8, 12.5]; non-bio-IR: UPA15, −0.3 [−7.3, 6.7]; UPA30, 6.4 [−2.9, 15.7]). The imbalance of the rates of herpes zoster in the upadacitinib groups versus placebo, as well as the dose effect, was more pronounced in the bio-IR subgroup compared to non-bio-IR subgroup. In the non-bio-IR subgroup, numerically higher rates of serious infections were observed in the UPA15 and UPA30 groups compared with placebo (non-bio-IR: UPA15, 5.1 [−2.4, 12.7]; UPA30, 4.9 [−2.5, 12.3]), while a modest increase in the rate was observed in the bio-IR subgroup with UPA30 over placebo (1.2 [−4.1, 6.4]). For the other AESIs, comparable rates were observed between upadacitinib and placebo in both bio-IR and non-bio-IR subgroups.

For UC, better outcomes were observed for UPA45 compared with placebo across the subgroups during induction (Figure S5). The adjusted differences between both doses of upadacitinib maintenance treatment and placebo were generally similar for efficacy outcomes across non-bio-IR, bio-IR, and TNF-IR subgroups (*clinical remission per adapted Mayo Score,* bio-IR: UPA15, 31.6; UPA30, 47.6; non-bio-IR: UPA15, 27.0; UPA30, 38.2; *maintenance of clinical response per adapted Mayo Score*, bio-IR: UPA15, 47.5; UPA30, 56.7; non-bio-IR: UPA15, 40.3; UPA30, 54.5; *endoscopic remission,* bio-IR: UPA15, 18.0; UPA30, 22.1; non-bio-IR: UPA15, 19.0; UPA30, 21.8; Figure 4 and Figure S6).

**Figure 5.**
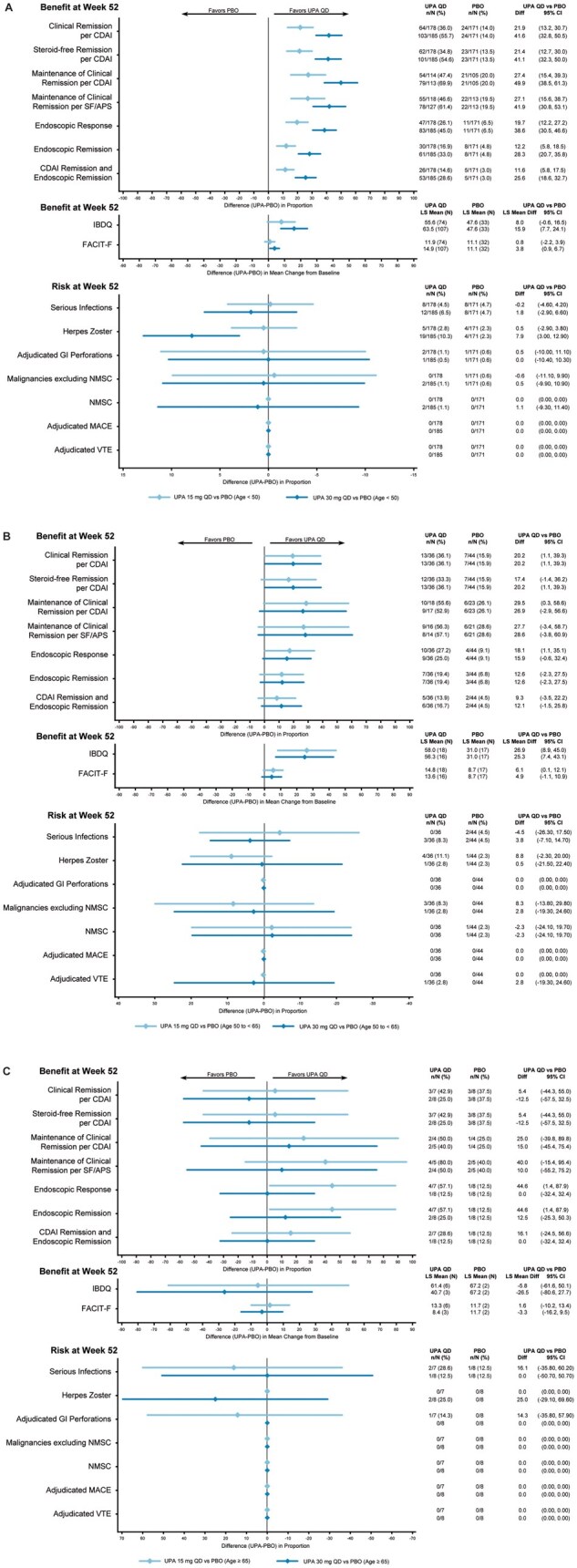
Benefit–risk during maintenance by age in patients with CD. (A) Patients aged <50 years. (B) Patients aged 50–64 years. (C) Patients aged ≥65 years. APS, abdominal pain score; CD, Crohn’s disease; CDAI, Crohn’s Disease Activity Index; FACIT-F, Functional Assessment of Chronic Illness Therapy-Fatigue; GI, gastrointestinal; IBDQ, Inflammatory Bowel Disease Questionnaire; LS, least squares; MACE, major adverse cardiovascular event; NMSC, ­nonmelanoma skin cancer; PBO, placebo; QD, once daily; SF, stool frequency; UPA, upadacitinib; VTE, venous thromboembolic event.

**Figure 6.**
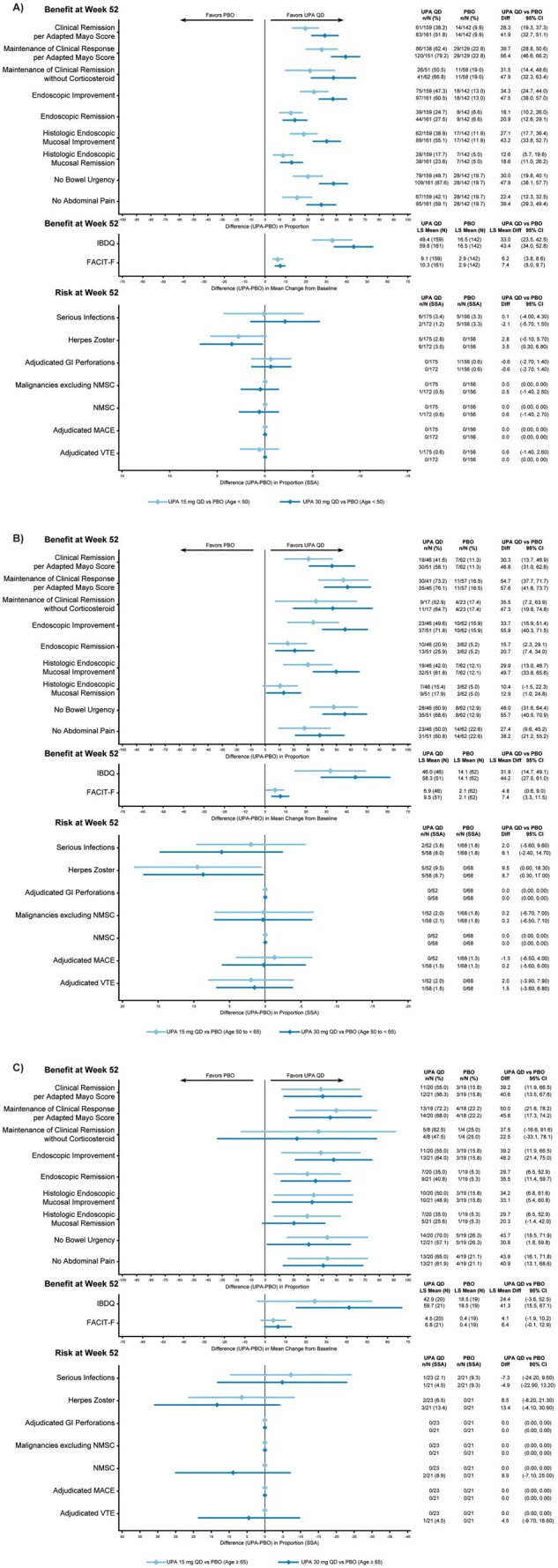
Benefit–risk during maintenance by age in patients with UC. (A) Patients aged <50 years. (B) Patients aged 50–64 years. (C) Patients aged ≥65 years. FACIT-F, Functional Assessment of Chronic Illness Therapy-Fatigue; GI, gastrointestinal; IBDQ, Inflammatory Bowel Disease Questionnaire; LS, least squares; MACE, major adverse cardiovascular event; NMSC, nonmelanoma skin cancer; PBO, placebo; QD, once daily; SSA, study-size adjusted; UC, ulcerative colitis; UPA, upadacitinib; VTE, venous thromboembolic event.

Overall, AESI rates for patients with UC were comparable between upadacitinib and placebo across the subgroups during the maintenance period, except for herpes zoster for which the imbalance versus placebo for UPA15 was more pronounced for the bio-IR group than for the non-bio-IR subgroup (bio-IR: UPA15, 6.2 [1.6, 10.8]; UPA30, 4.9 [0.6, 9.2]; non-bio-IR: UPA15, 3.1 [−0.6, 6.9]; UPA30, 6.2 [1.5, 10.9]).

#### Age

In CD, improved efficacy was observed with UPA45 versus placebo for all age subgroups (ages <50, 50–64, and ≥65 years; Figure S7) during the induction period, with greater improvements observed in patients <65 years. During the induction period for CD, numerically higher rates (response rate difference [95% CI]) of serious infections with upadacitinib versus placebo were seen in subgroups aged 50-64 and ≥65 years (50-64 years, 2.1 [−2.0, 6.1]; ≥65 years, 3.2 [−17.5, 24.0]), and for herpes zoster in the subgroups aged <65 years for UPA45 (<50 years, 1.8 [0.4, 3.2]; 50-64 years, 4.3 [−0.4, 9.0]).

During the maintenance period, patients who received UPA15 and UPA30 generally showed improved efficacy (response rate difference) in all age subgroups, with greater improvements observed in patients aged <65 years (*CDAI,* <50 years: UPA15, 21.9; UPA30, 41.6; 50-64 years: UPA15, 20.2; UPA30, 20.2; ≥65 years: UPA15, 5.4; UPA30, −12.5; *SF/APS,* <50 years*:* UPA15, 19.1; UPA30, 40.1; 50–64 years: UPA15, 15.2; UPA30, 15.2; ≥65 years: UPA15, 33.9; UPA30, −12.5; *endoscopic response,* <50 years: UPA15, 19.7; UPA30, 38.6; 50-64 years: UPA15, 18.1; UPA30, 15.9; ≥65 years: UPA15, 44.6; UPA30, 0; *endoscopic remission*, <50 years: UPA15, 12.2; UPA30, 28.3; 50-64 years: UPA15, 12.6; UPA30, 12.6; ≥65 years: UPA15, 44.6; UPA30, 12.5; Figure 5). In patients aged <50 years, UPA30 showed greater improvements than UPA15 when compared with placebo.

Generally, the rates of AESI among patients with CD during the maintenance period were low and comparable between upadacitinib and placebo, except for higher rates of herpes zoster with UPA30 in patients aged <50 years (7.9 [3.0, 12.9]).

For UC, UPA45 demonstrated greater improvements versus placebo after 8 weeks of induction treatment across the selected end points for all age subgroups (ages <50, 50-64, ≥65 years; Figure S8). During the induction period, the rates of AESI were generally low and similar for UPA45 and placebo.

During the maintenance period, patients showed greater efficacy improvements with UPA15 and UPA30 versus placebo across all clinical end points in all age subgroups by age (*clinical remission per adapted Mayo score*, <50 years: UPA15, 28.3; UPA30, 41.9; 50-64 years: UPA15, 30.3; UPA30, 46.8; ≥65 years: UPA15, 39.2; UPA30, 40.6; *maintenance of clinical response per adapted Mayo Score*, <50 years: UPA15, 39.7; UPA30, 56.4; 50-64 years: UPA15, 54.7; UPA30, 57.6; ≥65 years: UPA15, 50.0; UPA30, 45.8; *endoscopic remission*, <50 years: UPA15, 18.1; UPA30, 20.9; 50-64 years: UPA15, 15.7; UPA30, 20.7; ≥65 years: UPA15, 29.7; UPA30, 35.5; Figure 6).

There was generally a greater improvement with UPA30 in the subgroups of patients aged <65 years. During the maintenance period, AESI rates were generally low and comparable for UPA15, UPA30, and placebo, except for a numerically higher rate for serious infection with UPA30 in the groups aged 50-64 years (UPA15, 2.0 [−5.6, 9.6]; UPA30, 6.1 [−2.4, 14.7]), and a higher rate of herpes zoster with both upadacitinib maintenance doses in patients aged <50 years (UPA15, 2.8 [−0.1, 5.7]; UPA30, 3.5 [0.3, 6.8]).

#### Overall population

The benefit–risk profile for the overall CD and UC populations was favorable for upadacitinib versus placebo after 8–12 weeks of induction and 52 weeks of maintenance across the evaluated end points (Figure S9-S12). The benefits were generally more favorable for UPA30 compared with UPA15. The rates of AESI with upadacitinib were similar to that of placebo during the induction period, except for higher rates of herpes zoster with UPA45; Figure S9 and Figure S11). During the maintenance period, a numerically higher rate (response rate difference [95% CI]) of serious infections was observed in CD for UPA30 compared with placebo (2.1 [−2.3, 6.4]; Figure S10). A higher rate of herpes zoster was observed with both doses of upadacitinib maintenance treatment, most notably with UPA30 (*CD*: UPA15, 1.8 [−1.4, 5.1]; UPA30, 7.4 [3.1, 11.6]; *UC*: UPA15, 4.7 [1.9, 7.4]; UPA30, 5.6 [2.5, 8.6]; Figure S10 and Figure S12). MACE, VTE, malignancies (excluding NMSC), and gastrointestinal perforations were reported infrequently with rates comparable to placebo (Figure S10 and Figure S12).

## 4. Discussion

This post hoc analysis of data from CD and UC phase 2b/3 clinical trials evaluated the benefit–risk profile of the JAKi upadacitinib across the induction and maintenance periods of the upadacitinib clinical program overall and in demographic subgroups of CV risk status, prior treatment, and age. Previously, concerns emerged from phase 3 clinical trials of tofacitinib, a JAKi, regarding increased risks of MACE among patients with RA.^13^^,^^14^ These concerns were further supported by findings from the post-marketing ORAL Surveillance safety trial, which involved patients aged ≥50 years with RA and CV risk factors treated with tofacitinib.^10^ Findings from the trial suggested potential safety concerns, including an increased risk of herpes zoster, VTE, MACE, and malignancies.^10^ A pharmacovigilance study using the Food and Drug Administration’s Adverse Event Reporting System indicated an increased reporting of CV events such as strokes and ischemic heart disease among patients with RA treated with JAKis compared with anti-TNF therapies.^15^ Another analysis reported an increased risk of VTE and a numerically greater risk for MACE and serious infections with baricitinib (an oral selective JAK1/JAK2 inhibitor) in patients with RA.^16^ Conversely, several large-scale cohort studies of commercially approved JAKis did not find an increased risk of these adverse events in RA.^17–21^ A recent post hoc analysis spanning 16 clinical studies of RA, psoriatic arthritis, axial spondylarthritis, atopic dermatitis, and IBD, and including over 8600 patients, found that long-term treatment with upadacitinib was well-tolerated, with no evidence of a cumulative increase in safety risk with longer exposure.^22^ Additionally, a post hoc analysis of a phase 3 trial in patients with RA showed that UPA15 treatment generally resulted in comparable rates of adverse events, except for herpes zoster, and better efficacy outcomes compared with adalimumab, regardless of baseline CV risk status.^23^ Numerically higher rates of serious infections and NMSC were also observed with upadacitinib in the higher risk group.^23^ A consensus meeting with IBD physicians and CV experts in January 2023, based on a the results of a systematic literature search, concluded that available evidence from patients with IBD did not indicate a higher risk of CV events with JAKi treatment (including evidence from upadacitinib studies).^24^ Given these findings, the present analysis sought to better understand how the benefit–risk profile of upadacitinib in IBD may be impacted by factors such as CV risk, prior treatment, and age.

The benefit–risk profile of upadacitinib was favorable among subgroups of patients with CD and UC of low vs high CV risk, prior treatment failure (non-bio-IR, bio-IR, or TNF-IR), and by age during induction and maintenance of therapy, and comparable with the overall population. There was consistent clinical benefit within the subgroups of interest without increased safety risks. During the maintenance period, better clinical outcomes were observed across efficacy end points with both upadacitinib doses across CV risk groups, with patients receiving UPA30 showing numerically greater rates of improvement versus UPA15. The adjusted differences between both doses of upadacitinib maintenance treatment and placebo were generally similar for the efficacy outcomes across non-bio-IR, bio-IR, and TNF-IR subgroups, with numerically greater improvements with UPA30 in CD and UC. Efficacy outcomes were favorable for both UPA15 and UPA30 doses versus placebo across the selected end points in all age subgroups. There was a greater improvement with UPA30 among patients aged <50 years with CD and those aged <64 years with UC. Generally, the rates of AESI across subgroups were low and comparable between upadacitinib and placebo during induction and maintenance treatment for CD and UC, except for higher rates of herpes zoster and numerically higher rates of serious infections in CD, mainly observed with UPA30 during the maintenance period in in all subgroups, except for the group aged ≥65 years. However, the results in this group should be interpreted with caution due to the limited sample size.

Immunization for herpes zoster was low for the study populations included in this analysis, which could have contributed to the increased rates of this AESI reported here. The risk of herpes zoster has been shown in a systematic review and meta-analysis and consistently reported in patients with immunological diseases in response to treatment with JAKis, and guidelines have been proposed for the vaccination and management of this AESI.^25^^,^^26^ For other AESIs (MACE, VTE, malignancies [excluding NMSC], NMSC, and GI perforations), rates were low and comparable between upadacitinib and placebo in the overall populations of patients with CD and UC, as well as in the CV risk, prior therapy, and age subgroups.

The present study has several limitations. As a post hoc analysis with end points not predefined and small patient numbers in the subgroups, the results should be considered exploratory, warranting further research. A post hoc analysis of the ORAL Surveillance study revealed differences in risk factors across geographical regions.^27^ A similar future analysis of these studies would be of interest. The limited number of patients in certain subgroups, particularly those aged ≥65 years, adds complexity to data interpretation, as the small sample size hinders comprehensive conclusions about the anticipated increase in CV risk and safety events in this population. Furthermore, the present analysis covers slightly more than 1 year of treatment, which may be insufficient to capture adverse events or risks that usually have longer latency, such as MACE and malignancies. For example, the ORAL Surveillance study had a median follow-up time of 4 years to evaluate MACE and malignancies.^10^ Hence, longer-term studies should provide a basis for more definitive conclusions about the benefit–risk profile of upadacitinib. It should be noted, however, that the safety profile of upadacitinib through 3 years of long-term extension treatment is consistent with the findings reported here.^28^ Additionally, the inclusion and exclusion criteria often employed in randomized clinical trials may not fully reflect the heterogeneity and diversity of real-world populations, thereby affecting the generalizability of the study findings.

This analysis provides important insights into the benefits and risks of upadacitinib for the treatment of IBD in important subgroups based on CV risk, prior treatment failure, and age. Though there was no difference in efficacy expected across subgroups, these data further underscore the efficacy of upadacitinib for the treatment of CD and UC, regardless of CV risk, prior treatment failure, and age. The integrated approach of this analysis allows for a comprehensive assessment of the efficacy and safety of upadacitinib across multiple studies involving both CD and UC. These findings add to the body of evidence on the efficacy and safety of upadacitinib and will enable clinicians and patients to make better informed treatment decisions.

In conclusion, this post hoc analysis of CV risk, prior treatment, and age in subgroups of patients with CD and UC during upadacitinib induction and maintenance therapy demonstrates that the benefits and risks were in general comparable to the previously reported results in the overall populations of patients with CD and UC. Our findings suggest the favorable benefit–risk profile of upadacitinib for the treatment of moderately to severely active CD and UC in general, and for the specific subgroups evaluated.

## Supplementary Material

jjaf198_Supplementary_Data

## Data Availability

AbbVie is committed to responsible data sharing regarding the clinical trials we sponsor. This includes access to anonymized, individual, and trial-level data (analysis data sets), as well as other information (e.g., protocols, clinical study reports, or analysis plans), as long as the trials are not part of an ongoing or planned regulatory submission. This includes requests for clinical trial data for unlicensed products and indications. These clinical trial data can be requested by any qualified researchers who engage in rigorous, independent, scientific research, and will be provided following review and approval of a research proposal, Statistical Analysis Plan (SAP), and execution of a Data Sharing Agreement (DSA). Data requests can be submitted at any time after approval in the United States and Europe and after acceptance of this manuscript for publication. The data will be accessible for 12 months, with possible extensions considered. For more information on the process or to submit a request, visit the following link: https://vivli.org/ourmember/abbvie/ then select “Home.”
