# Supplementary material for: Benefit–risk profile of upadacitinib: exploratory post hoc analysis of phase 2b/3 studies in patients with moderately to severely active ulcerative colitis or Crohn’s disease
Source: J Crohns Colitis. 2025 Nov 20;20(1):jjaf198. doi: 10.1093/ecco-jcc/jjaf198 (PMC12795599; doi:10.1093/ecco-jcc/jjaf198)
Supplement: jjaf198_Supplementary_Data [file jjaf198_supplementary_data.pdf]

Supplementary Information

Benefit-Risk Profile of Upadacitinib: Exploratory Post Hoc Analysis of Phase 2b/3 Studies in Patients With Moderately to Severely Active Ulcerative Colitis or Crohn's Disease

Severine Vermeire[1]; Jean-Frederic Colombel[2]; Silvio Danese[3]; Remo Panaccione[4]; Laurent Peyrin-Biroulet[5,6]; Kendall Beck[7]; María Chaparro[8]; Javier P. Gisbert[8]; Elena Dubcenco[9]; Justin Klaff[9]; Grace Naling[9]; Sharanya Ford[9]; Valencia Remple[9]; Namita Joshi[9]; Smitha Suravaram[9]; Benjamin Duncan[9]; Yibo Wang[9], Bettina Wick-Urban[10]; Edward V. Loftus, Jr[11]

[1]Department of Gastroenterology and Hepatology, University Hospitals of Leuven, Leuven, Belgium; [2]Henry D. Janowitz Division of Gastroenterology, Department of Medicine, Icahn School of Medicine at Mount Sinai, New York, NY, USA; [3]Gastroenterology and Endoscopy Unit, IRCCS Ospedale San Raffaele, Milan, Italy; [4]Inflammatory Bowel Disease Unit, Division of Gastroenterology and Hepatology, University of Calgary, Calgary, Alberta, Canada; [5]Department of Gastroenterology, CHRU Nancy, INSERM NGERE, Université de Lorraine, F-54500 Vandœuvre-lès-Nancy, France; [6]Division of Gastroenterology and Hepatology, McGill University Health Centre, Montreal, Quebec, Canada; [7]Division of Gastroenterology and Hepatology, Department of Medicine, University of California, San Francisco, CA, USA; [8]Gastroenterology Unit, Hospital Universitario de La Princesa, Instituto de Investigación Sanitaria Princesa (IIS-Princesa), Universidad Autónoma de Madrid (UAM), Centro de Investigación Biomédica en Red de Enfermedades Hepáticas y Digestivas (CIBEREHD), Madrid, Spain; [9]AbbVie Inc., North Chicago, IL, USA; [10]AbbVie Deutschland GmbH & Co. KG, Ludwigshafen, Germany; [11]Division of Gastroenterology and Hepatology, Mayo Clinic College of Medicine and Science, Rochester, MN, USA

Appendix 1. Outcome Definitions

Table S1. Demographics and clinical characteristics at induction baseline.

Figure S1. Benefit-risk during induction by cardiovascular risk factors in patients with Crohn's disease. (A) Low CV risk factors. (B) High CV risk factors.

Figure S2. Benefit-risk during induction by cardiovascular risk factors in patients with ulcerative colitis.

Figure S3. Benefit-risk during induction by prior biologic or TNF response status in patients with Crohn's disease.

Figure S4. Benefit-risk during maintenance by prior inadequate response to anti-TNF in patients with Crohn's disease.

Figure S5. Benefit-risk during induction by prior biologic or TNF response status in patients with ulcerative colitis.

Figure S6. Benefit-risk during maintenance by prior inadequate response to anti-TNF in patients with ulcerative colitis.

Figure S7. Benefit-risk during induction by age (< 50, 50–64, ≥ 65 years) in patients with Crohn's disease.

Figure S8. Benefit-risk during induction by age (< 50, 50–64, ≥ 65 years) in patients with ulcerative colitis.

Figure S9. Benefit-risk during induction in the overall population of patients with Crohn's disease.

Figure S10. Benefit-risk during maintenance in the overall population of patients with Crohn's disease.

Figure S11. Benefit-risk during induction in the overall population of patients with ulcerative colitis.

Figure S12. Benefit-risk during maintenance in the overall population of patients with ulcerative colitis.

Appendix 1. Outcome Definitions

For Crohn's disease (CD), the outcomes assessed were the Crohn's Disease Activity Index (CDAI) clinical remission (defined as CDAI < 150), stool frequency/abdominal pain Score (SF/APS) clinical remission (defined as daily very soft or liquid SF ≤ 2.8 and average daily APS ≤ 1.0, with neither worse than baseline), maintenance of clinical remission (per CDAI or SF/APS), glucocorticoid-free clinical remission (per CDAI or SF/APS), clinical response by the CDAI score (defined as a decrease of ≥ 100 points from baseline [CR-100]), endoscopic response (defined as a decrease in Simple Endoscopic Score for Crohn's Disease [SES-CD] > 50% from baseline [or for patients with baseline SES-CD of 4, ≥ 2-point reduction from baseline]), endoscopic remission (defined as SES-CD ≤ 4 and ≥ 2-point reduction from baseline and no subscore > 1 in any individual variable, as scored by central reviewer), deep remission (clinical remission and endoscopic remission), change from baseline in the Inflammatory Bowel Disease Questionnaire (IBDQ) questionnaire, and change from baseline in fatigue (Functional Assessment of Chronic Illness Therapy-Fatigue [FACIT-Fatigue]).

For UC, the selected endpoints were clinical remission per Adapted Mayo Score (defined as stool frequency subscore [SFS] ≤ 1 and not greater than baseline, rectal bleeding subscore [RBS] = 0, and endoscopic subscore ≤ 1 without friability), maintenance of clinical response per Adapted Mayo Score (defined as a decrease ≥ 2 and ≥ 30% from baseline, plus a decrease in RBS ≥ 1 or an absolute RBS ≤ 1) at week 52 among patients who achieved clinical response per Adapted Mayo Score at the end of the induction period, maintenance of clinical remission per Adapted Mayo Score (defined as clinical remission at week 52 among patients who achieved clinical remission per Adapted Mayo Score at the end of induction), corticosteroid-free clinical remission per Adapted Mayo Score (defined as clinical remission and corticosteroid-free for ≥ 90 days immediately before week 52 among patients who achieved clinical remission per Adapted Mayo Score at the end of induction), endoscopic improvement (defined as an endoscopic subscore ≤ 1 without friability), endoscopic remission (defined as an endoscopic subscore = 0), histological endoscopic mucosal improvement (defined as an endoscopic subscore ≤ 1 without friability and Geboes score ≤ 3.1),

mucosal healing (also known as histological endoscopic mucosal remission defined as an endoscopic subscore = 0 and Geboes score < 2), no abdominal pain (mean score of 0 across 3 days), no bowel urgency (mean score of 0 across 3 days), change from baseline in FACIT-Fatigue score, and change from baseline in IBDQ.

Table S1. Patient demographics and clinical characteristics at induction baseline.

	Ulcerative Colitis		Crohn's Disease	
**Parameter, n (%)**	**Placebo (N = 378)**	**UPA 45 mg QD (N = 719)**	**Placebo (N = 347)**	**UPA 45 mg QD (N = 674)**
Female	142 (37.6)	268 (37.3)	157 (45.2)	316 (46.9)
Age, years, mean (SD)	43.1 (14.4)	42.6 (14.4)	38.4 (12.9)	39.1 (13.7)
Age group, years				
< 50	252 (66.7)	482 (67.0)	275 (79.3)	505 (74.9)
50–64	93 (24.6)	175 (24.3)	63 (18.2)	139 (20.6)
≥ 65	33 (8.7)	62 (8.6)	9 (2.6)	30 (4.5)
Race				
White	265 (70.1)	481 (66.9)	256 (73.8)	488 (72.4)
Black or African American	10 (2.6)	24 (3.3)	10 (2.9)	36 (5.3)
Asian	95 (25.1)	205 (28.5)	74 (21.3)	142 (21.1)
American Indian or Alaska Native	3 (0.8)	0	1 (0.3)	1 (0.1)
Native Hawaiian or Other Pacific Islander	1 (0.3)	1 (0.1)	0	0
Multiple	4 (1.1)	8 (1.1)	6 (1.7)	7 (1.0)
BMI, kg/m^2, mean (SD)	25.5 (6.2)	24.8 (5.3)	24.8 (6.6)	24.3 (6.0)
BMI				
< 30	307 (81.2)	615 (85.5)	287 (82.7)	563 (83.5)
≥ 30	70 (18.5)	101 (14.0)	60 (17.3)	111 (16.5)
Missing	1 (0.3)	3 (0.4)	0	0
Years since diagnosis, mean (SD)	8.2 (8.0)	7.9 (6.8)	9.5 (8.1)	10.6 (9.6)

Adapted Mayo Score, mean (SD)	7.0 (1.2)	7.0 (1.2)	–	–
CDAI, mean (SD)	–	–	300.9 (85.0)	299.2 (85.5)
SES-CD, mean (SD)	–	–	14.3 (7.4)	14.4 (7.6)
SES-CD				
< 15	–	–	204 (58.8)	399 (59.2)
≥ 15	–	–	143 (41.2)	275 (40.8)
CV risk factors				
Any CV risk factor	228 (60.3)	435 (60.5)	228 (65.7)	428 (63.5)
Aged ≥ 65 years	33 (8.7)	62 (8.6)	9 (2.6)	30 (4.5)
Black or African American	10 (2.6)	24 (3.3)	10 (2.9)	36 (5.3)
BMI ≥ 30 kg/m^2	70 (18.5)	101 (14.0)	60 (17.3)	111 (16.5)
Current tobacco smoker	25 (6.6)	72 (10.0)	77 (22.2)	136 (20.2)
Tobacco smoker within the past 15 years	84 (22.2)	146 (20.3)	46 (13.3)	99 (14.7)
Current alcohol consumption ≥ 4 drinks/day	5 (1.3)	13 (1.8)	1 (0.3)	6 (0.9)
History of cardiovascular disease	23 (6.1)	43 (6.0)	10 (2.9)	24 (3.6)
History of diabetes mellitus	20 (5.3)	36 (5.0)	7 (2.0)	19 (2.8)
History of hypertension	60 (15.9)	110 (15.3)	32 (9.2)	91 (13.5)
History of VTE	10 (2.6)	11 (1.5)	5 (1.4)	14 (2.1)
History of chronic kidney disease	4 (1.1)	3 (0.4)	0	6 (0.9)
Prior antihypertensive use	1 (0.3)	3 (0.4)	0	3 (0.4)
Decreased HDL cholesterol (< 40 mg/dL)	57 (15.1)	111 (15.4)	89 (25.6)	129 (19.1)
Elevated blood pressure	26 (6.9)	48 (6.7)	19 (5.5)	36 (5.3)
Previous biologic history				
Without failure	174 (46.0)	334 (46.5)	99 (28.5)	189 (28.0)
With failure	204 (54.0)	385 (53.5)	248 (71.5)	485 (72.0)
> 1 biologic	138 (36.5)	251 (34.9)	161 (46.4)	316 (46.9)
≥ 1 Anti-TNF failure	76 (20.1)	125 (17.4)	99 (28.5)	202 (30.0)

Herpes Zoster vaccination by age group, years	8 (2.1)	30 (4.2)	17 (4.9)	45 (6.7)
< 50	4 (1.1)	16 (2.2)	12 (3.5)	22 (3.3)
50–64	3 (0.8)	5 (0.7)	4 (1.2)	15 (2.2)
≥ 65	1 (0.3)	9 (1.3)	1 (0.3)	8 (1.2)

BMI, body mass index; CDAI, Crohn's Disease Activity Index; SES-CD, Simple Endoscopic Score for Crohn's Disease; TNF, tumor necrosis factor; VTE, venous thromboembolism.

Figure S1. Benefit-risk during induction by cardiovascular risk factors in patients with Crohn's disease.

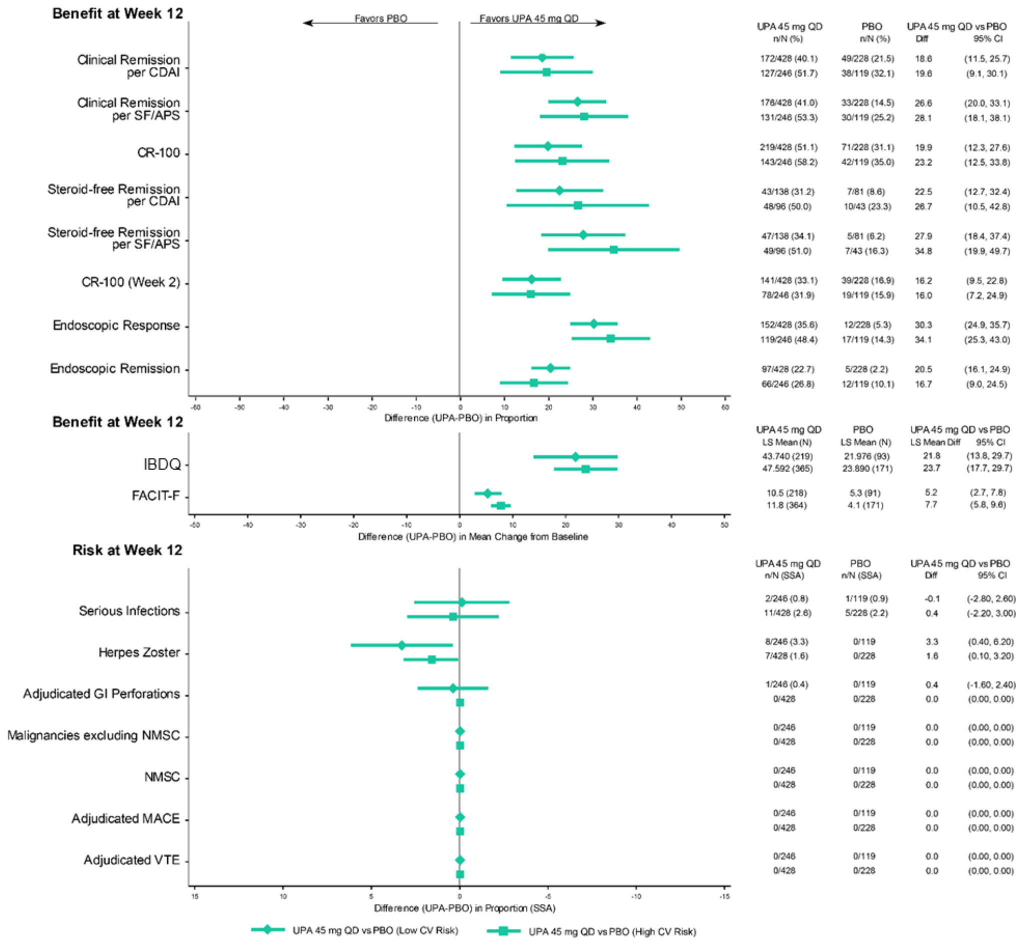

APS, abdominal pain score; CD, Crohn's disease; CDAI, Crohn's Disease Activity Index; CR-100, clinical response by the CDAI score (decrease of ≥ 100 points from baseline); CV, cardiovascular; FACIT-F, Functional Assessment of Chronic Illness Therapy-Fatigue; IBDQ, Inflammatory Bowel Disease Questionnaire; LS, least squares; MACE, major adverse cardiovascular event; NMSC, nonmelanoma skin cancer; PBO, placebo; QD, once daily; SF, stool frequency; SSA, study-size adjusted; UPA, upadacitinib; VTE, venous thromboembolic event.

CV risk factors in this analysis used to classify patients as high risk included age ≥ 65 years, Black or African American race, BMI ≥ 30 mg/kg, current or former (within 15 years) tobacco smoker, excess alcohol use (> 4 drinks/day), a history of CV disease or other relevant cardiac problems, diabetes mellitus, hypertension, VTE/thrombosis of limbs or major organs, chronic kidney disease, baseline or prior hypertensive use, decreased HDL cholesterol (< 40 mg/dL), or elevated blood pressure. A patient meeting at least one of the previously identified CV risk factors are designated as high CV risk, whereas all other patients are designated as low CV risk.

Figure S2. Benefit-risk during induction by cardiovascular risk factors in patients with ulcerative colitis.

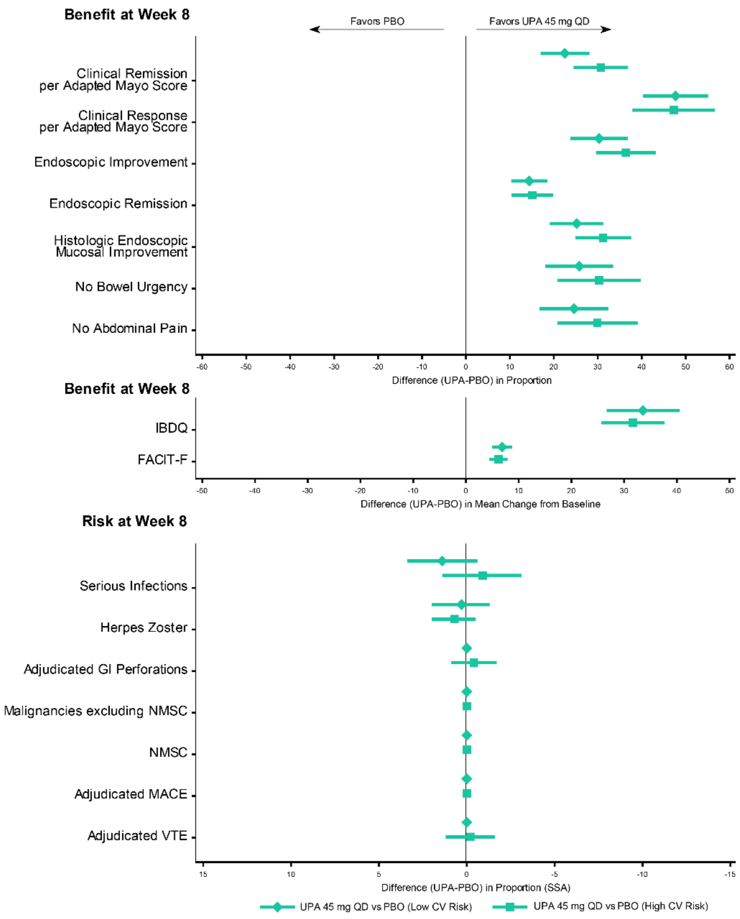

UPA 45 mg QD n/N (%)	PBO n/N (%)	UPA 45 mg QD vs PBO Diff	UPA 45 mg QD vs PBO 95% CI
114/400 (28.4)	12/201 (6.1)	22.3	(16.8, 27.9)
84/260 (32.3)	2/127 (1.8)	30.5	(24.3, 36.7)
292/400 (72.9)	51/201 (25.4)	47.5	(40.1, 54.9)
194/260 (74.6)	35/127 (27.6)	47.1	(37.7, 56.5)
163/400 (40.8)	21/201 (10.7)	30.1	(23.6, 36.6)
103/260 (39.6)	4/127 (3.4)	36.2	(29.4, 43.0)
65/400 (16.2)	4/201 (2.0)	14.2	(10.1, 18.3)
41/260 (15.8)	1/127 (0.8)	14.9	(10.2, 19.7)
134/400 (33.5)	17/201 (8.5)	25.0	(18.9, 31.1)
87/260 (33.5)	3/127 (2.4)	31.0	(24.7, 37.4)
200/400 (50.0)	49/201 (24.4)	25.6	(17.9, 33.3)
138/260 (52.9)	29/127 (22.8)	30.1	(20.6, 39.6)
205/400 (51.3)	54/201 (26.9)	24.4	(16.5, 32.2)
126/260 (48.6)	24/127 (18.9)	29.7	(20.6, 38.9)

UPA 45 mg QD LS Mean (N)	PBO LS Mean (N)	UPA 45 mg QD vs PBO LS Mean Diff	UPA 45 mg QD vs PBO 95% CI
52.2 (243)	18.8 (109)	33.4	(26.5, 40.3)
54.2 (364)	22.7 (172)	31.5	(25.5, 37.5)
9.9 (242)	3.2 (109)	6.7	(4.8, 8.6)
9.0 (361)	3.0 (171)	6.0	(4.2, 7.7)

UPA 45 mg QD n/N (SSA)	PBO n/N (SSA)	UPA 45 mg QD vs PBO Diff	UPA 45 mg QD vs PBO 95% CI
4/284 (1.4)	0/150	1.4	(-0.60, 3.40)
5/435 (1.2)	5/228 (2.1)	-0.9	(-3.10, 1.40)
1/284 (0.3)	0/150	0.3	(-1.30, 2.00)
3/435 (0.7)	0/228	0.7	(-0.50, 2.00)
0/284	0/150	0.0	(0.00, 0.00)
0/435	1/228 (0.4)	-0.4	(-1.70, 0.90)
0/284	0/150	0.0	(0.00, 0.00)
0/435	0/228	0.0	(0.00, 0.00)
0/284	0/150	0.0	(0.00, 0.00)
0/435	0/228	0.0	(0.00, 0.00)
0/284	0/150	0.0	(0.00, 0.00)
0/435	0/228	0.0	(0.00, 0.00)
0/284	0/150	0.0	(0.00, 0.00)
1/435 (0.3)	1/228 (0.4)	-0.2	(-1.60, 1.20)

CV, cardiovascular; FACIT-F, Functional Assessment of Chronic Illness Therapy-Fatigue; IBDQ, Inflammatory Bowel Disease Questionnaire; LS, least squares; MACE, major adverse cardiovascular event; NMSC, nonmelanoma skin cancer; PBO, placebo; QD, once daily; SSA, study-size adjusted; UC, ulcerative colitis; UPA, upadacitinib; VTE, venous thromboembolic event.

CV risk factors in this analysis used to classify patients as high risk included age ≥ 65 years, Black or African American race, BMI ≥ 30 mg/kg, current or former (within 15 years) tobacco smoker, excess alcohol use (> 4 drinks/day), a history of CV disease or other relevant cardiac problems, diabetes mellitus, hypertension, VTE/thrombosis of limbs or major organs, chronic kidney disease, baseline or prior hypertensive use, decreased HDL cholesterol (< 40 mg/dL), or elevated blood pressure. A patient meeting at least one of the previously identified CV risk factors are designated as high CV risk, whereas all other patients are designated as low CV risk.

Figure S3. Benefit-risk during induction by prior biologic or TNF response status in patients with Crohn's disease.

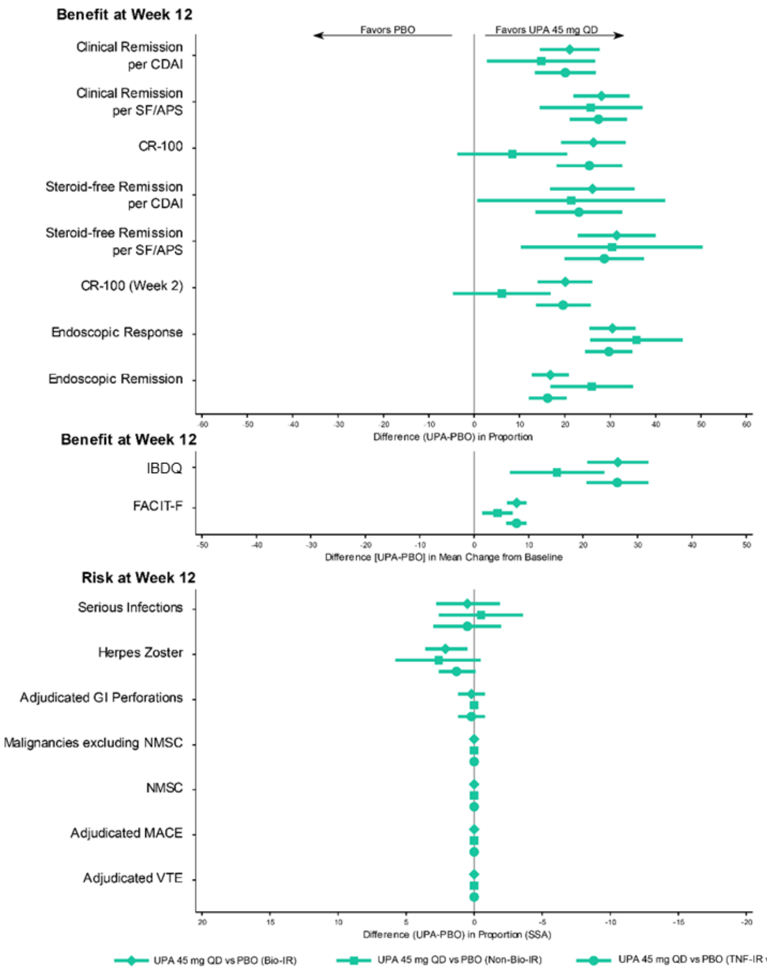

	UPA 45 mg QD n/N (%)	PBO n/N (%)	UPA 45 mg QD vs PBO Diff	UPA 45 mg QD vs PBO 95% CI
Clinical Remission per CDAI	197/485 (40.5)	48/248 (19.4)	21.1	(14.5, 27.7)
	102/189 (54.1)	39/99 (39.4)	14.8	(2.8, 26.7)
	184/465 (39.5)	46/239 (19.3)	20.1	(13.4, 26.8)
Clinical Remission per SF/APS	205/485 (42.2)	35/248 (14.1)	28.1	(21.9, 34.3)
	102/189 (54.0)	28/99 (28.3)	25.7	(14.4, 37.1)
	192/465 (41.2)	33/239 (13.8)	27.4	(21.1, 33.7)
CR-100	258/485 (53.2)	67/248 (26.9)	26.3	(19.2, 33.4)
	104/189 (54.9)	46/99 (46.5)	8.4	(-3.7, 20.5)
	244/465 (52.5)	65/239 (27.1)	25.4	(18.2, 32.7)
Steroid-free Remission per CDAI	65/178 (36.5)	10/96 (10.5)	26.1	(16.7, 35.4)
	26/56 (46.4)	7/28 (25.0)	21.4	(0.7, 42.1)
	58/170 (34.1)	10/91 (11.0)	23.1	(13.5, 32.7)
Steroid-free Remission per SF/APS	67/178 (37.6)	6/96 (6.3)	31.4	(22.8, 40.0)
	29/56 (51.8)	6/28 (21.4)	30.4	(10.3, 50.4)
	60/170 (35.3)	6/91 (6.6)	28.7	(19.9, 37.5)
CR-100 (Week 2)	162/485 (33.4)	33/248 (13.3)	20.1	(14.0, 26.1)
	58/189 (30.7)	24/99 (24.6)	6.1	(-4.7, 16.9)
	152/465 (32.6)	31/239 (13.0)	19.6	(13.6, 25.7)
Endoscopic Response	173/485 (35.7)	13/248 (5.3)	30.5	(25.4, 35.6)
	98/189 (52.0)	16/99 (16.2)	35.8	(25.6, 46.0)
	163/465 (35.1)	13/239 (5.5)	29.7	(24.4, 34.9)
Endoscopic Remission	95/485 (19.6)	7/248 (2.8)	16.8	(12.7, 20.9)
	68/189 (36.0)	10/99 (10.1)	25.9	(16.8, 35.0)
	89/465 (19.1)	7/239 (2.9)	16.2	(12.1, 20.4)

	UPA 45 mg QD LS Mean (N)	PBO LS Mean (N)	UPA 45 mg QD vs PBO LS Mean Diff	UPA 45 mg QD vs PBO 95% CI
IBDQ	46.644 (420)	20.228 (186)	26.4	(20.8, 32.1)
	46.424 (164)	31.191 (78)	15.2	(6.5, 23.9)
	45.933 (402)	19.634 (179)	26.3	(20.6, 32.0)
FACIT-F	11.6 (418)	3.8 (185)	7.8	(6.0, 9.6)
	11.5 (164)	7.2 (77)	4.3	(1.5, 7.1)
	11.4 (400)	3.7 (178)	7.8	(5.9, 9.6)

	UPA 45 mg QD n/N (SSA)	PBO n/N (SSA)	UPA 45 mg QD vs PBO Diff	UPA 45 mg QD vs PBO 95% CI
Serious Infections	12/485 (2.5)	5/248 (2.0)	0.5	(-1.90, 2.80)
	1/189 (0.5)	1/99 (1.0)	-0.5	(-3.60, 2.60)
	12/465 (2.6)	5/239 (2.1)	0.5	(-2.00, 3.00)
Herpes Zoster	10/485 (2.1)	0/248	2.1	(0.50, 3.60)
	5/189 (2.6)	0/99	2.6	(-0.50, 5.80)
	6/465 (1.3)	0/239	1.3	(-0.10, 2.60)
Adjudicated GI Perforations	1/485 (0.2)	0/248	0.2	(-0.80, 1.20)
	0/189	0/99	0.0	(0.00, 0.00)
	1/465 (0.2)	0/239	0.2	(-0.80, 1.20)
Malignancies excluding NMSC	0/485	0/248	0.0	(0.00, 0.00)
	0/189	0/99	0.0	(0.00, 0.00)
	0/465	0/239	0.0	(0.00, 0.00)
NMSC	0/485	0/248	0.0	(0.00, 0.00)
	0/189	0/99	0.0	(0.00, 0.00)
	0/465	0/239	0.0	(0.00, 0.00)
Adjudicated MACE	0/485	0/248	0.0	(0.00, 0.00)
	0/189	0/99	0.0	(0.00, 0.00)
	0/465	0/239	0.0	(0.00, 0.00)
Adjudicated VTE	0/485	0/248	0.0	(0.00, 0.00)
	0/189	0/99	0.0	(0.00, 0.00)
	0/465	0/239	0.0	(0.00, 0.00)

APS, abdominal pain score; CD, Crohn's disease; CDAI, Crohn's Disease Activity Index; CR-100, clinical response by the CDAI score (decrease of ≥ 100 points from baseline); FACIT-F, Functional Assessment of Chronic Illness Therapy-Fatigue; IBDQ, Inflammatory Bowel Disease Questionnaire; LS, least squares; MACE, major adverse cardiovascular event; NMSC, nonmelanoma skin cancer; PBO, placebo; QD, once daily; SF, stool frequency; SSA, study-size adjusted; TNF, tumor necrosis factor; UPA, upadacitinib; VTE, venous thromboembolic event.

TNF-IR is defined as patients with a previous inadequate response to anti-TNF therapy (TNF-IR) within the bio-IR population. Bio-IR is defined as patients with an inadequate response, loss of response, or intolerance to a biologic.

Figure S4. Benefit-risk during maintenance by prior inadequate response to anti-TNF in patients with Crohn's disease.

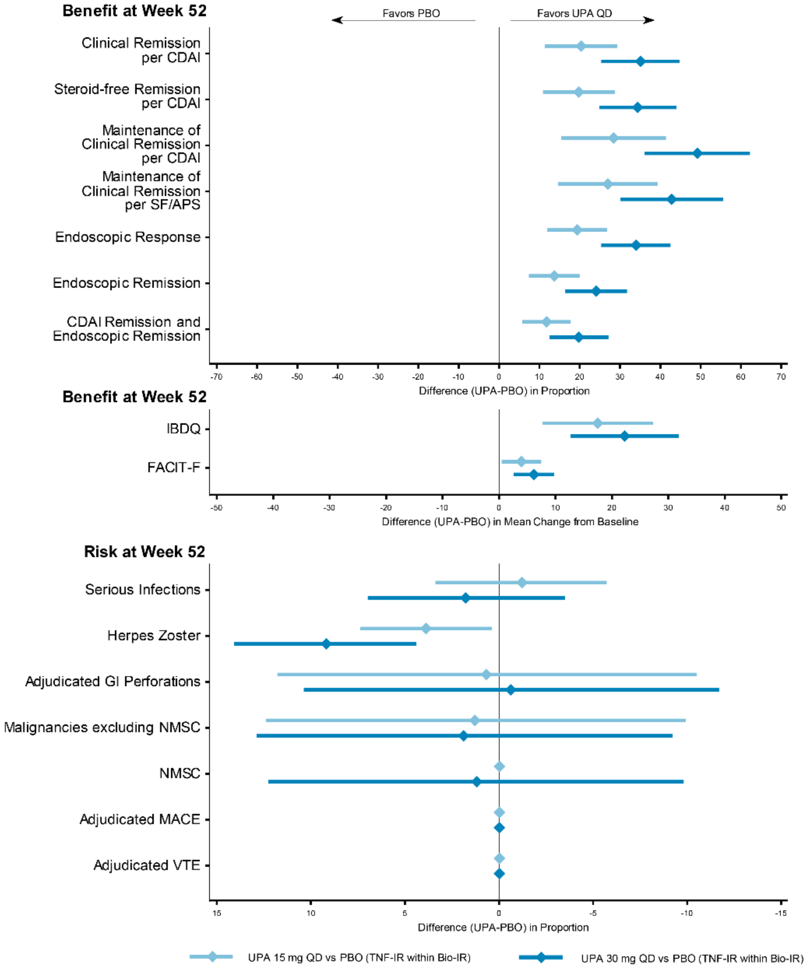

Benefit at Week 52

Outcome	UPA QD n/N (%)	PBO n/N (%)	UPA QD vs PBO Diff	UPA QD vs PBO 95% CI
Clinical Remission per CDAI	51/155 (32.9)	20/159 (12.6)	20.3	(11.3, 29.3)
	77/162 (47.5)	20/159 (12.6)	35.0	(25.7, 44.2)
Steroid-free Remission per CDAI	49/155 (31.6)	19/159 (11.9)	19.7	(10.8, 28.6)
	75/162 (46.3)	19/159 (11.9)	34.3	(25.2, 43.5)
Maintenance of Clinical Remission per CDAI	41/90 (45.6)	15/87 (17.2)	28.3	(15.3, 41.3)
	59/89 (66.3)	15/87 (17.2)	49.1	(36.4, 61.7)
Maintenance of Clinical Remission per SF/APS	40/94 (42.6)	15/96 (15.6)	26.9	(14.6, 39.3)
	56/96 (58.3)	15/96 (15.6)	42.7	(30.5, 55.0)
Endoscopic Response	37/155 (23.7)	7/159 (4.4)	19.3	(11.8, 26.7)
	62/162 (38.3)	7/159 (4.4)	33.9	(25.7, 42.0)
Endoscopic Remission	25/155 (16.1)	4/159 (2.5)	13.6	(7.3, 19.9)
	43/162 (26.5)	4/159 (2.5)	24.0	(16.8, 31.2)
CDAI Remission and Endoscopic Remission	22/155 (14.2)	4/159 (2.5)	11.7	(5.7, 17.7)
	36/162 (22.2)	4/159 (2.5)	19.7	(12.9, 26.6)

Benefit at Week 52

Outcome	UPA QD LS Mean (N)	PBO LS Mean (N)	UPA QD vs PBO LS Mean Diff	UPA QD vs PBO 95% CI
IBDQ	57.0 (58)	39.6 (28)	17.4	(7.6, 27.2)
	61.8 (88)	39.6 (28)	22.2	(12.9, 31.5)
FACIT-F	13.2 (58)	9.3 (28)	3.9	(0.4, 7.4)
	15.5 (88)	9.3 (28)	6.1	(2.8, 9.4)

Risk at Week 52

Outcome	UPA QD n/N (%)	PBO n/N (%)	UPA QD vs PBO Diff	UPA QD vs PBO 95% CI
Serious Infections	6/155 (3.9)	8/159 (5.0)	-1.2	(-5.70, 3.40)
	11/162 (6.8)	8/159 (5.0)	1.8	(-3.40, 6.90)
Herpes Zoster	7/155 (4.5)	1/159 (0.6)	3.9	(0.40, 7.40)
	16/162 (9.9)	1/159 (0.6)	9.2	(4.50, 14.00)
Adjudicated GI Perforations	2/155 (1.3)	1/159 (0.6)	0.7	(-10.50, 11.80)
	0/162	1/159 (0.6)	-0.6	(-11.60, 10.30)
Malignancies excluding NMSC	2/155 (1.3)	0/159	1.3	(-9.90, 12.40)
	3/162 (1.9)	0/159	1.9	(-9.10, 12.80)
NMSC	0/155	0/159	0.0	(0.00, 0.00)
	2/162 (1.2)	0/159	1.2	(-9.70, 12.20)
Adjudicated MACE	0/155	0/159	0.0	(0.00, 0.00)
	0/162	0/159	0.0	(0.00, 0.00)
Adjudicated VTE	0/155	0/159	0.0	(0.00, 0.00)
	0/162	0/159	0.0	(0.00, 0.00)

APS, abdominal pain score; CD, Crohn's disease; CDAI, Crohn's Disease Activity Index; FACIT-F, Functional Assessment of Chronic Illness Therapy-Fatigue; IBDQ, Inflammatory Bowel Disease Questionnaire; LS, least squares; MACE, major adverse cardiovascular event; NMSC, nonmelanoma skin cancer; PBO, placebo; QD, once daily; SF, stool frequency; TNF, tumor necrosis factor; UPA, upadacitinib; VTE, venous thromboembolic event.

TNF-IR is defined as patients with a previous inadequate response to anti-TNF therapy (TNF-IR) within the bio-IR population. Bio-IR is defined as patients with an inadequate response, loss of response, or intolerance to a biologic.

Figure S5. Benefit-risk during induction by prior biologic or TNF response status in patients with ulcerative colitis.

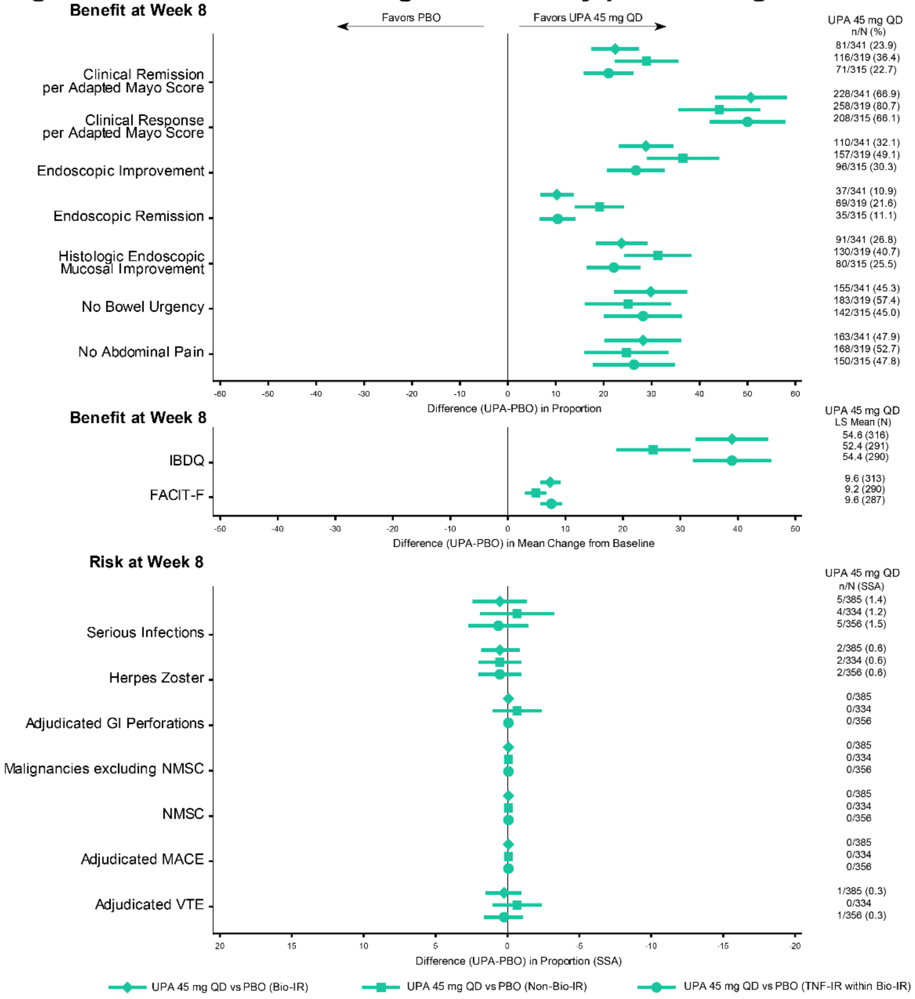

	UPA 45 mg QD	PBO	UPA 45 mg QD vs PBO	
	n/N (%)	n/N (%)	Diff	95% CI
Clinical Remission per Adapted Mayo Score	81/341 (23.9)	2/167 (1.5)	22.4	(17.4, 27.4)
	116/319 (36.4)	12/161 (7.5)	28.9	(22.3, 35.6)
	71/315 (22.7)	2/149 (1.7)	21.0	(15.8, 26.2)
Clinical Response per Adapted Mayo Score	228/341 (66.9)	27/167 (16.2)	50.7	(43.2, 58.2)
	258/319 (80.7)	59/161 (36.6)	44.1	(35.5, 52.7)
	208/315 (66.1)	24/149 (16.1)	50.0	(42.1, 57.9)
Endoscopic Improvement	110/341 (32.1)	6/167 (3.3)	28.8	(23.1, 34.5)
	157/319 (49.1)	20/161 (12.5)	36.5	(29.0, 44.1)
	96/315 (30.3)	6/149 (3.7)	26.7	(20.7, 32.7)
Endoscopic Remission	37/341 (10.9)	1/167 (0.7)	10.2	(6.7, 13.7)
	69/319 (21.6)	4/161 (2.5)	19.1	(13.9, 24.2)
	35/315 (11.1)	1/149 (0.7)	10.4	(6.6, 14.1)
Histologic Endoscopic Mucosal Improvement	91/341 (26.8)	5/167 (3.1)	23.7	(18.3, 29.1)
	130/319 (40.7)	15/161 (9.4)	31.3	(24.2, 38.3)
	80/315 (25.5)	5/149 (3.4)	22.1	(16.4, 27.7)
No Bowel Urgency	155/341 (45.3)	26/167 (15.6)	29.8	(22.1, 37.4)
	183/319 (57.4)	52/161 (32.3)	25.1	(16.0, 34.1)
	142/315 (45.0)	25/149 (16.8)	28.2	(20.0, 36.3)
No Abdominal Pain	163/341 (47.9)	33/167 (19.8)	28.2	(20.1, 36.2)
	168/319 (52.7)	45/161 (28.0)	24.7	(15.9, 33.5)
	150/315 (47.8)	32/149 (21.5)	26.3	(17.7, 34.9)

	UPA 45 mg QD	PBO	UPA 45 mg QD vs PBO	
	LS Mean (N)	LS Mean (N)	LS Mean Diff	95% CI
IBDQ	54.6 (316)	15.5 (144)	39.1	(32.8, 45.4)
	52.4 (291)	27.0 (137)	25.4	(19.0, 31.9)
	54.4 (290)	15.3 (127)	39.1	(32.3, 46.0)
FACIT-F	9.6 (313)	2.1 (144)	7.5	(5.7, 9.3)
	9.2 (290)	4.2 (136)	5.0	(3.1, 6.9)
	9.6 (287)	2.0 (127)	7.7	(5.8, 9.6)

	UPA 45 mg QD	PBO	UPA 45 mg QD vs PBO	
	n/N (SSA)	n/N (SSA)	Diff	95% CI
Serious Infections	5/385 (1.4)	2/204 (0.8)	0.6	(-1.30, 2.50)
	4/334 (1.2)	3/174 (1.8)	-0.6	(-3.20, 2.00)
	5/356 (1.5)	2/184 (0.8)	0.7	(-1.40, 2.80)
Herpes Zoster	2/385 (0.6)	0/204	0.6	(-0.80, 1.90)
	2/334 (0.6)	0/174	0.6	(-0.90, 2.10)
	2/356 (0.6)	0/184	0.6	(-0.90, 2.10)
Adjudicated GI Perforations	0/385	0/204	0.0	(0.00, 0.00)
	0/334	1/174 (0.6)	-0.6	(-2.30, 1.10)
	0/356	0/184	0.0	(0.00, 0.00)
Malignancies excluding NMSC	0/385	0/204	0.0	(0.00, 0.00)
	0/334	0/174	0.0	(0.00, 0.00)
	0/356	0/184	0.0	(0.00, 0.00)
NMSC	0/385	0/204	0.0	(0.00, 0.00)
	0/334	0/174	0.0	(0.00, 0.00)
	0/356	0/184	0.0	(0.00, 0.00)
Adjudicated MACE	0/385	0/204	0.0	(0.00, 0.00)
	0/334	0/174	0.0	(0.00, 0.00)
	0/356	0/184	0.0	(0.00, 0.00)
Adjudicated VTE	1/385 (0.3)	0/204	0.3	(-0.90, 1.60)
	0/334	1/174 (0.6)	-0.6	(-2.30, 1.10)
	1/356 (0.3)	0/184	0.3	(-1.00, 1.70)

FACIT-F, Functional Assessment of Chronic Illness Therapy-Fatigue; IBDQ, Inflammatory Bowel Disease Questionnaire; LS, least squares; MACE, major adverse cardiovascular event; NMSC, nonmelanoma skin cancer; PBO, placebo; QD, once daily; SSA, study-size adjusted; TNF, tumor necrosis factor; UC, ulcerative colitis; UPA, upadacitinib; VTE, venous thromboembolic event.

TNF-IR is defined as patients with a previous inadequate response to anti-TNF therapy (TNF-IR) within the bio-IR population. Bio-IR is defined as patients with an inadequate response, loss of response, or intolerance to a biologic.

Figure S6. Benefit-risk during maintenance by prior inadequate response to anti-TNF in patients with ulcerative colitis.

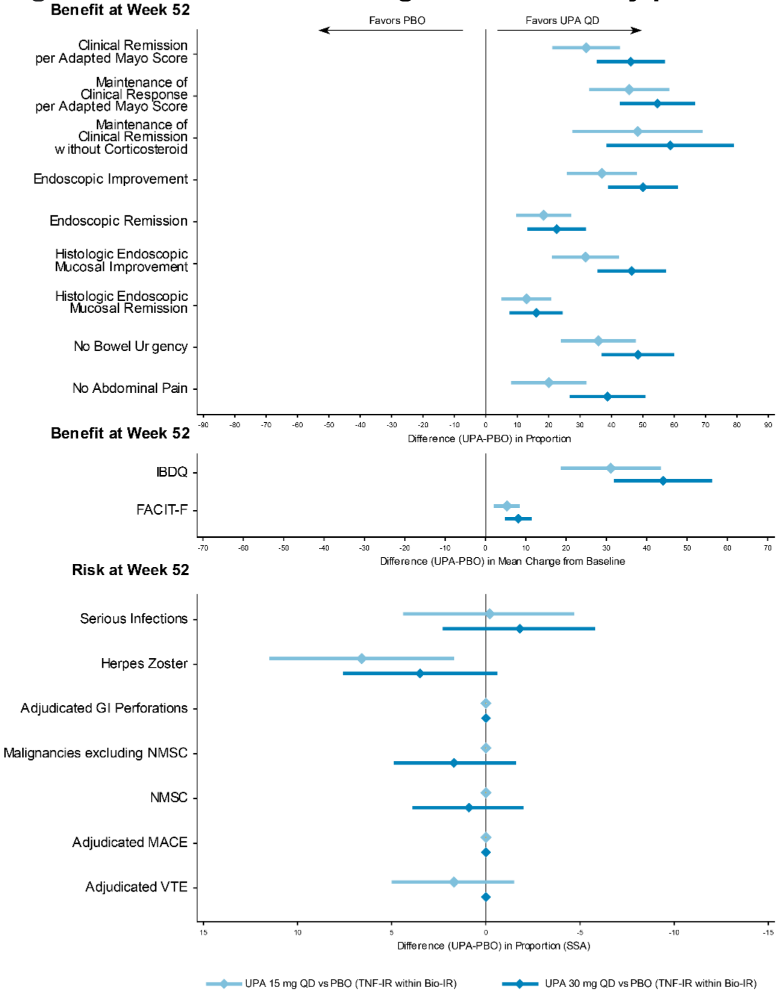

Benefit at Week 52

	UPA QD	PBO	UPA QD vs PBO	
	n/N (%)	n/N (%)	Diff	95% CI
Clinical Remission per Adapted Mayo Score	39/101 (38.7)	7/102 (6.9)	31.9	(21.1, 42.7)
	55/103 (52.9)	7/102 (6.9)	46.1	(35.2, 57.0)
Maintenance of Clinical Response per Adapted Mayo Score	53/86 (61.6)	14/90 (16.0)	45.6	(32.8, 58.4)
	66/94 (70.6)	14/90 (16.0)	54.6	(42.6, 66.6)
Maintenance of Clinical Remission without Corticosteroid	19/32 (59.4)	3/27 (11.1)	48.3	(27.5, 69.0)
	22/31 (69.8)	3/27 (11.1)	58.7	(38.4, 79.0)
Endoscopic Improvement	45/101 (44.4)	8/102 (7.4)	36.9	(25.8, 48.0)
	59/103 (57.4)	8/102 (7.4)	50.0	(38.9, 61.1)
Endoscopic Remission	22/101 (21.4)	3/102 (3.1)	18.3	(9.5, 27.1)
	26/103 (25.6)	3/102 (3.1)	22.5	(13.2, 31.8)
Histologic Endoscopic Mucosal Improvement	38/101 (37.9)	6/102 (6.2)	31.7	(21.0, 42.4)
	54/103 (52.6)	6/102 (6.2)	46.4	(35.5, 57.4)
Histologic Endoscopic Mucosal Remission	16/101 (15.9)	3/102 (3.1)	12.9	(4.9, 20.8)
	20/103 (19.1)	3/102 (3.1)	16.0	(7.5, 24.4)
No Bowel Urgency	51/101 (50.5)	15/102 (14.7)	35.8	(23.9, 47.7)
	65/103 (63.1)	15/102 (14.7)	48.4	(36.8, 60.0)
No Abdominal Pain	38/101 (37.6)	18/102 (17.6)	20.0	(8.0, 32.0)
	58/103 (56.3)	18/102 (17.6)	38.7	(26.6, 50.8)

Benefit at Week 52

	UPA QD	PBO	UPA QD vs PBO	
	LS Mean (N)	LS Mean (N)	LS Mean Diff	95% CI
IBDQ	43.8 (101)	12.6 (102)	31.1	(18.7, 43.6)
	56.7 (103)	12.6 (102)	44.1	(31.9, 56.2)
FACIT-F	6.7 (101)	1.3 (102)	5.4	(2.1, 8.6)
	9.5 (103)	1.3 (102)	8.2	(4.9, 11.5)

Risk at Week 52

	UPA QD	PBO	UPA QD vs PBO	
	n/N (SSA)	n/N (SSA)	Diff	95% CI
Serious Infections	3/117 (2.4)	3/117 (2.6)	-0.2	(-4.70, 4.40)
	1/114 (0.8)	3/117 (2.6)	-1.8	(-5.80, 2.30)
Herpes Zoster	8/117 (6.6)	0/117	6.6	(1.70, 11.50)
	4/114 (3.5)	0/117	3.5	(-0.60, 7.60)
Adjudicated GI Perforations	0/117	0/117	0.0	(0.00, 0.00)
	0/114	0/117	0.0	(0.00, 0.00)
Malignancies excluding NMSC	0/117	0/117	0.0	(0.00, 0.00)
	2/114 (1.7)	0/117	1.7	(-1.60, 4.90)
NMSC	0/117	0/117	0.0	(0.00, 0.00)
	1/114 (0.9)	0/117	0.9	(-2.00, 3.90)
Adjudicated MACE	0/117	0/117	0.0	(0.00, 0.00)
	0/114	0/117	0.0	(0.00, 0.00)
Adjudicated VTE	2/117 (1.7)	0/117	1.7	(-1.50, 5.00)
	0/114	0/117	0.0	(0.00, 0.00)

FACIT-F, Functional Assessment of Chronic Illness Therapy-Fatigue; IBDQ, Inflammatory Bowel Disease Questionnaire; LS, least squares; MACE, major adverse cardiovascular event; NMSC, nonmelanoma skin cancer; PBO, placebo; QD, once daily; SSA, study-size adjusted; TNF, tumor necrosis factor; UC, ulcerative colitis; UPA, upadacitinib; VTE, venous thromboembolic event.

TNF-IR is defined as patients with a previous inadequate response to anti-TNF therapy (TNF-IR) within the bio-IR population. Bio-IR is defined as patients with an inadequate response, loss of response, or intolerance to a biologic.

Figure S7. Benefit-risk during induction by age (< 50, 50– to < 645, ≥ 65 years) in patients with Crohn's disease.

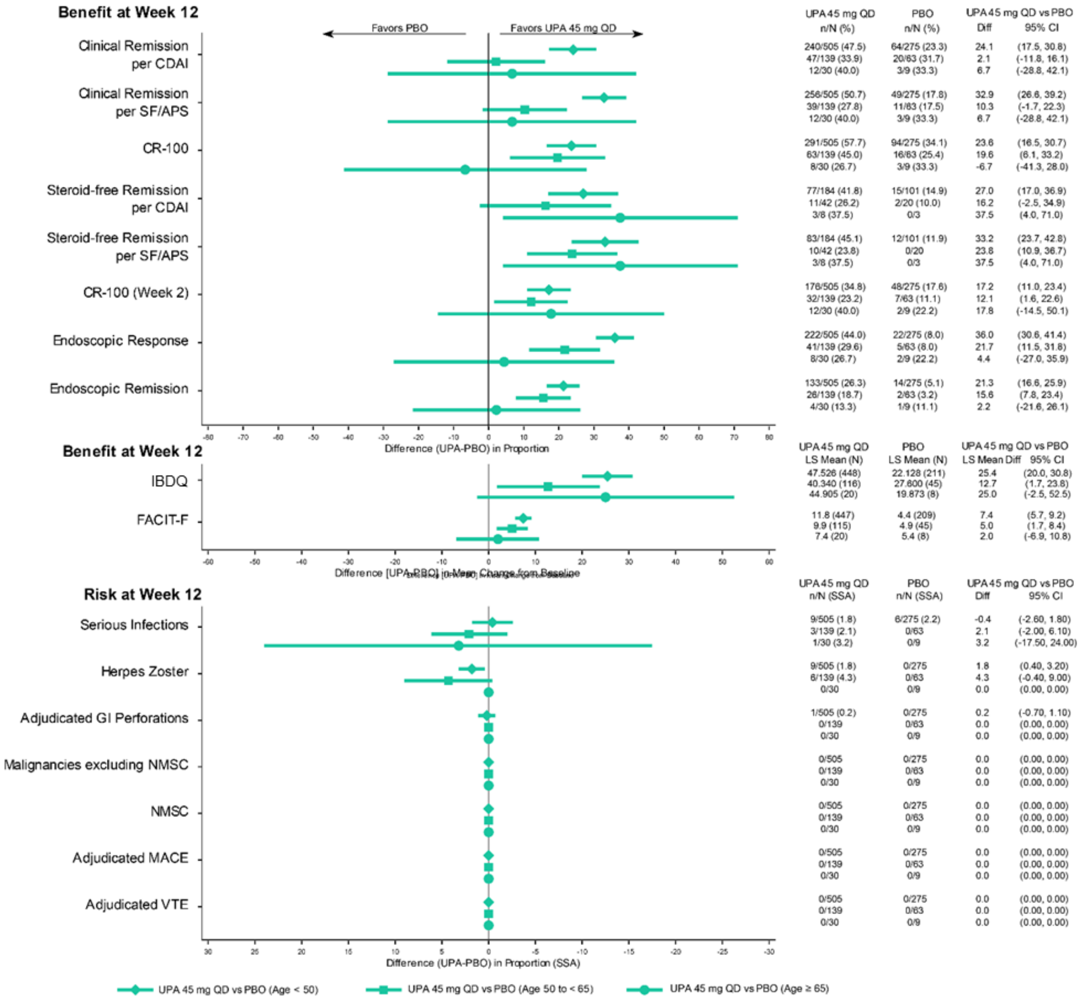

APS, abdominal pain score; CD, Crohn's disease; CDAI, Crohn's Disease Activity Index; CR-100, clinical response by the CDAI score (decrease of ≥ 100 points from baseline); FACIT-F, Functional Assessment of Chronic Illness Therapy-Fatigue; IBDQ, Inflammatory Bowel Disease Questionnaire; LS, least squares; MACE, major adverse cardiovascular event; NMSC, nonmelanoma skin cancer; PBO, placebo; QD, once daily; SF, stool frequency; SSA, study-size adjusted; UPA, upadacitinib; VTE, venous thromboembolic event.

Figure S8. Benefit-risk during induction by age (< 50, 50–64, ≥ 65 years) in patients with ulcerative colitis.

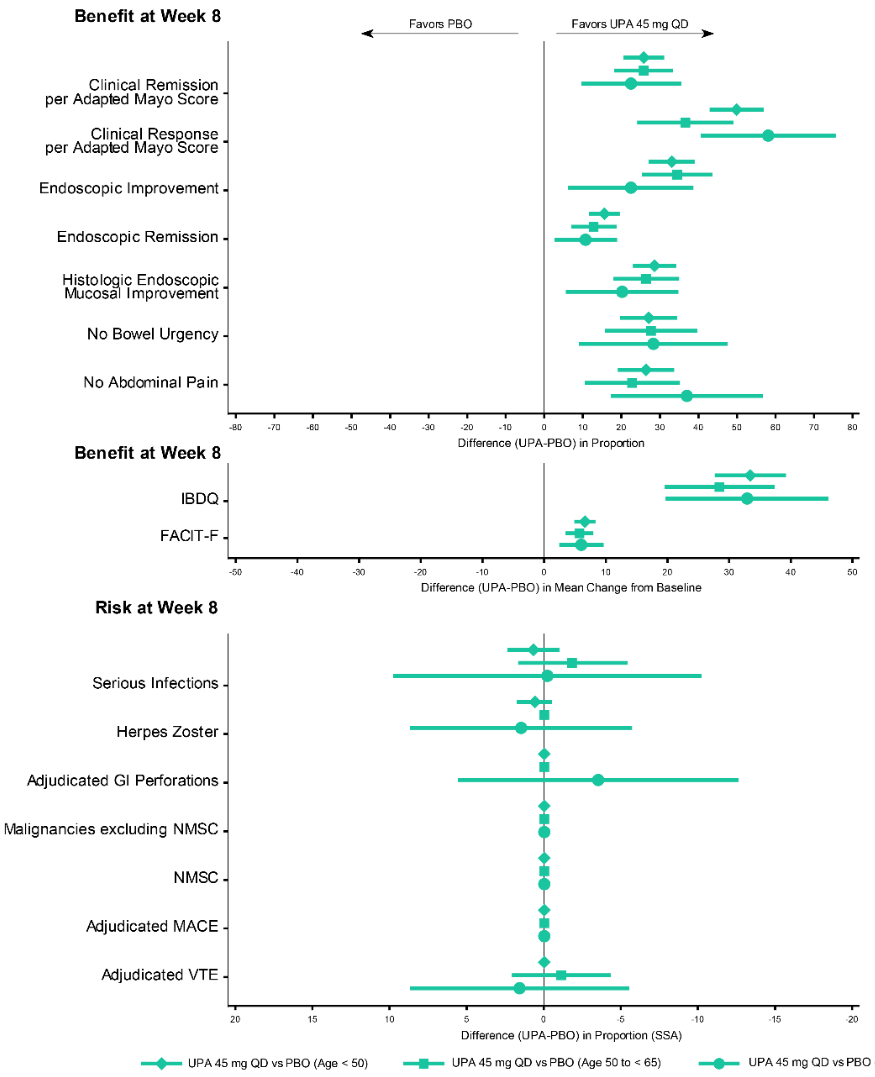

	UPA 45 mg QD n/N (%)	PBO n/N (%)	UPA 45 mg QD vs PBO Diff	UPA 45 mg QD vs PBO 95% CI
Clinical Remission per Adapted Mayo Score	136/436 (31.1)	11/217 (5.3)	25.8	(20.5, 31.1)
	47/166 (28.2)	2/81 (2.5)	25.8	(18.1, 33.4)
	15/58 (25.9)	1/30 (3.3)	22.5	(9.6, 35.5)
Clinical Response per Adapted Mayo Score	324/436 (74.3)	53/217 (24.4)	49.9	(42.9, 56.9)
	118/166 (71.2)	28/81 (34.7)	36.6	(24.1, 49.1)
	43/58 (74.8)	5/30 (16.7)	58.1	(40.6, 75.6)
Endoscopic Improvement	180/436 (41.2)	18/217 (8.2)	33.1	(27.1, 39.0)
	68/166 (40.7)	5/81 (6.3)	34.5	(25.3, 43.7)
	19/58 (32.5)	3/30 (10.0)	22.5	(6.2, 38.7)
Endoscopic Remission	76/436 (17.5)	4/217 (1.9)	15.6	(11.6, 19.6)
	23/166 (14.1)	1/81 (1.2)	12.8	(7.0, 18.7)
	6/58 (10.7)	0/30	10.7	(2.6, 18.9)
Histologic Endoscopic Mucosal Improvement	153/436 (35.2)	14/217 (6.6)	28.6	(23.0, 34.2)
	52/166 (31.4)	4/81 (4.9)	26.4	(17.9, 35.0)
	16/58 (26.8)	2/30 (6.7)	20.2	(5.6, 34.8)
No Bowel Urgency	225/436 (51.5)	53/217 (24.4)	27.1	(19.7, 34.5)
	85/166 (51.2)	19/81 (23.5)	27.7	(15.8, 39.7)
	28/58 (48.3)	6/30 (20.0)	28.3	(9.0, 47.5)
No Abdominal Pain	213/436 (49.0)	49/217 (22.6)	26.4	(19.1, 33.7)
	83/166 (50.0)	22/81 (27.2)	22.8	(10.5, 35.2)
	35/58 (60.3)	7/30 (23.3)	37.0	(17.3, 56.7)

	UPA 45 mg QD LS Mean (N)	PBO LS Mean (N)	UPA 45 mg QD vs PBO LS Mean Diff	UPA 45 mg QD vs PBO 95% CI
IBDQ	54.2 (400)	20.7 (190)	33.4	(27.6, 39.2)
	50.2 (152)	21.7 (66)	28.4	(19.5, 37.4)
	57.5 (55)	24.6 (25)	32.9	(19.6, 46.1)
FACIT-F	10.2 (398)	3.6 (190)	6.6	(4.9, 8.3)
	8.4 (150)	2.7 (66)	5.7	(3.5, 8.0)
	6.6 (55)	0.6 (24)	6.0	(2.4, 9.6)

	UPA 45 mg QD n/N (SSA)	PBO n/N (SSA)	UPA 45 mg QD vs PBO Diff	UPA 45 mg QD vs PBO 95% CI
Serious Infections	7/482 (1.5)	2/252 (0.7)	0.7	(-1.00, 2.40)
	0/175	2/93 (1.8)	-1.8	(-5.40, 1.70)
	2/62 (3.3)	1/33 (3.5)	-0.2	(-10.20, 9.80)
Herpes Zoster	3/482 (0.6)	0/252	0.6	(-0.50, 1.80)
	0/175	0/93	0.0	(0.00, 0.00)
	1/62 (1.5)	0/33	1.5	(-5.70, 8.70)
Adjudicated GI Perforations	0/482	0/252	0.0	(0.00, 0.00)
	0/175	0/93	0.0	(0.00, 0.00)
	0/62	1/33 (3.5)	-3.5	(-12.60, 5.60)
Malignancies excluding NMSC	0/482	0/252	0.0	(0.00, 0.00)
	0/175	0/93	0.0	(0.00, 0.00)
	0/62	0/33	0.0	(0.00, 0.00)
NMSC	0/482	0/252	0.0	(0.00, 0.00)
	0/175	0/93	0.0	(0.00, 0.00)
	0/62	0/33	0.0	(0.00, 0.00)
Adjudicated MACE	0/482	0/252	0.0	(0.00, 0.00)
	0/175	0/93	0.0	(0.00, 0.00)
	0/62	0/33	0.0	(0.00, 0.00)
Adjudicated VTE	0/482	0/252	0.0	(0.00, 0.00)
	0/175	1/93 (1.1)	-1.1	(-4.30, 2.10)
	1/62 (1.6)	0/33	1.6	(-5.50, 8.70)

FACIT-F, Functional Assessment of Chronic Illness Therapy-Fatigue; IBDQ, Inflammatory Bowel Disease Questionnaire; LS, least squares; MACE, major adverse cardiovascular event; NMSC, nonmelanoma skin cancer; PBO, placebo; QD, once daily; SSA, study-size adjusted; UC, ulcerative colitis; UPA, upadacitinib; VTE, venous thromboembolic event.

Figure S9. Benefit-risk during induction in the overall population of patients with Crohn's disease.

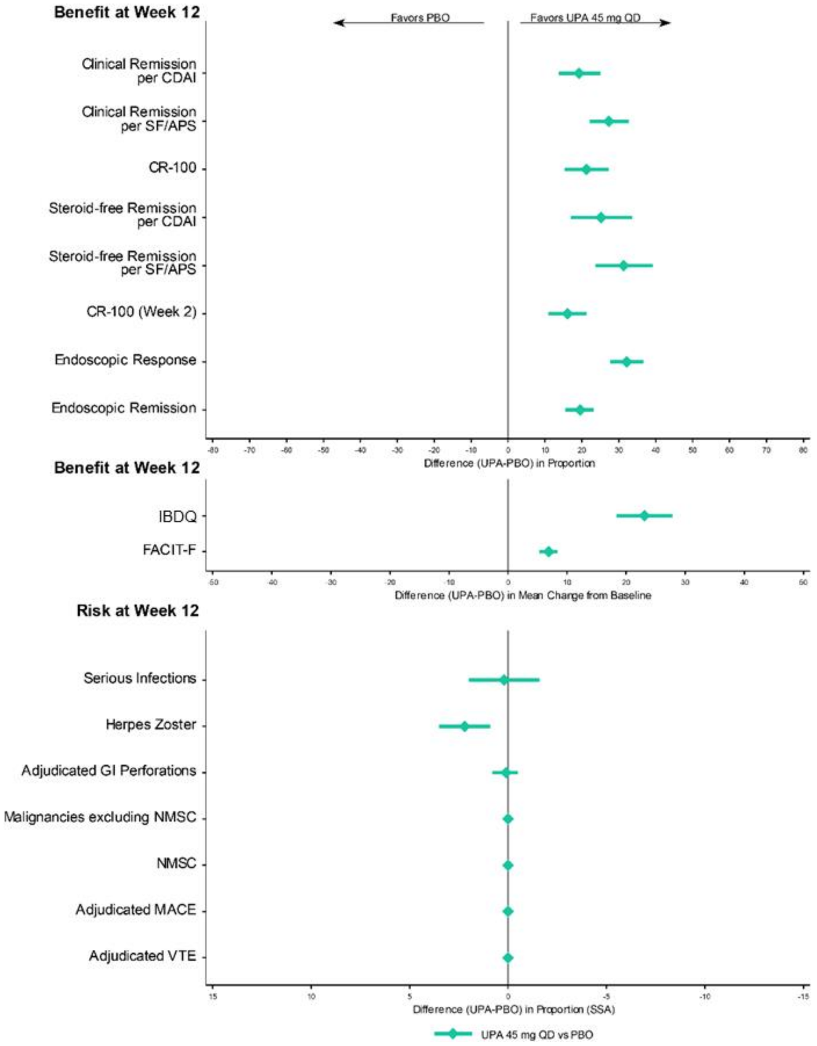

Benefit at Week 12

	UPA 45 mg QD n/N (%)	PBO n/N (%)	UPA 45 mg QD vs PBO Diff	95% CI
Clinical Remission per CDAI	299/674 (44.3)	87/347 (25.1)	19.2	(13.7, 25.0)
Clinical Remission per SF/APS	307/674 (45.5)	63/347 (18.2)	27.3	(22.1, 32.7)
CR-100	362/674 (53.7)	113/347 (32.5)	21.2	(15.3, 27.3)
Steroid-free Remission per CDAI	91/234 (38.9)	17/124 (13.7)	25.2	(17.0, 33.6)
Steroid-free Remission per SF/APS	96/234 (41.0)	12/124 (9.7)	31.3	(23.7, 39.2)
CR-100 (Week 2)	220/674 (32.6)	57/347 (16.5)	16.1	(10.9, 21.2)
Endoscopic Response	271/674 (40.3)	29/347 (8.4)	31.9	(27.6, 36.7)
Endoscopic Remission	163/674 (24.2)	17/347 (4.9)	19.3	(15.5, 23.2)

Benefit at Week 12

	UPA 45 mg QD LS Mean (N)	PBO LS Mean (N)	UPA 45 mg QD vs PBO LS Mean Diff	95% CI
IBDQ	46.080 (584)	22.953 (264)	23.1	(18.4, 27.9)
FACIT-F	11.4 (582)	4.5 (262)	6.9	(5.3, 8.4)

Risk at Week 12

	UPA 45 mg QD n/N (SSA)	PBO n/N (SSA)	UPA 45 mg QD vs PBO Diff	95% CI
Serious Infections	13/674 (1.9)	6/347 (1.7)	0.2	(-1.60, 2.00)
Herpes Zoster	15/674 (2.2)	0/347	2.2	(0.90, 3.50)
Adjudicated GI Perforations	1/674 (0.1)	0/347	0.1	(-0.50, 0.80)
Malignancies excluding NMSC	0/674	0/347	0.0	(0.00, 0.00)
NMSC	0/674	0/347	0.0	(0.00, 0.00)
Adjudicated MACE	0/674	0/347	0.0	(0.00, 0.00)
Adjudicated VTE	0/674	0/347	0.0	(0.00, 0.00)

APS, abdominal pain score; CD, Crohn's disease; CDAI, Crohn's Disease Activity Index; CR-100, clinical response by the CDAI score (decrease of ≥ 100 points from baseline); FACIT-F, Functional Assessment of Chronic Illness Therapy-Fatigue; IBDQ, Inflammatory Bowel Disease Questionnaire; LS, least squares; MACE, major adverse cardiovascular event; NMSC, nonmelanoma skin cancer; PBO, placebo; QD, once daily; SF, stool frequency; SSA, study-size adjusted; UPA, upadacitinib; VTE, venous thromboembolic event.

Figure S10. Benefit-risk during maintenance in the overall population of patients with Crohn's disease.

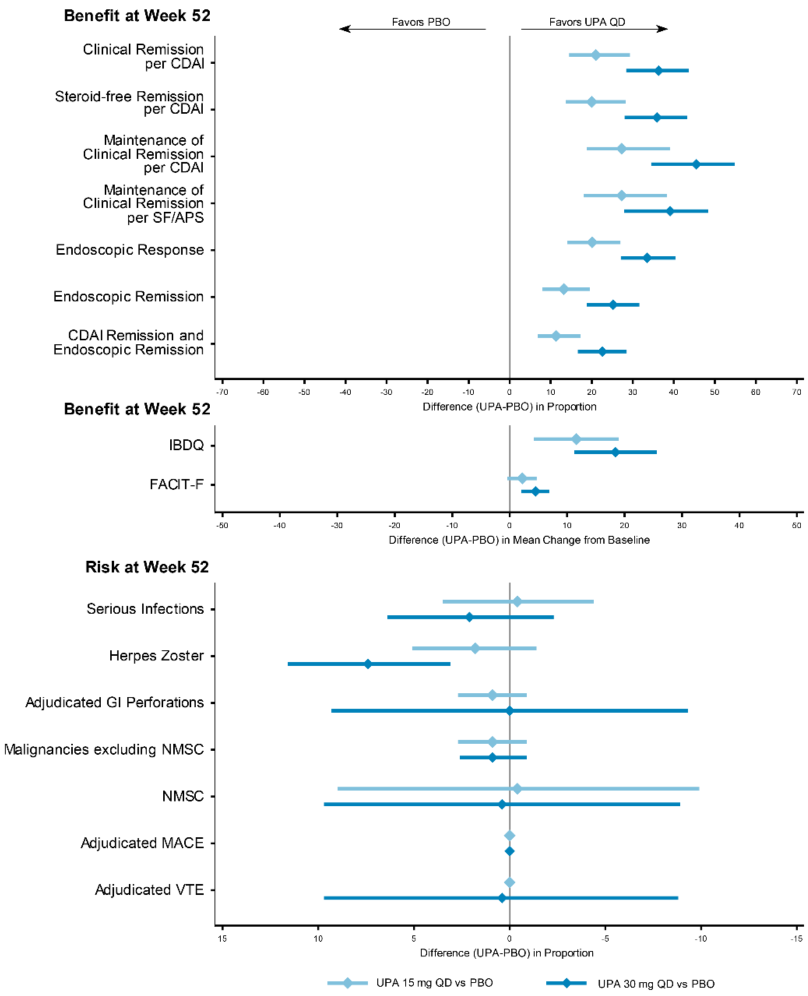

Benefit at Week 52

	UPA QD n/N (%)	PBO n/N (%)	UPA QD vs PBO Diff	UPA QD vs PBO 95% CI
Clinical Remission per CDAI	80/221 (36.2)	34/223 (15.2)	21.0	(14.5, 29.3)
	118/229 (51.5)	34/223 (15.2)	36.3	(28.4, 43.7)
Steroid-free Remission per CDAI	77/221 (34.8)	33/223 (14.8)	20.0	(13.7, 28.3)
	116/229 (50.7)	33/223 (14.8)	35.9	(28.0, 43.3)
Maintenance of Clinical Remission per CDAI	66/136 (48.5)	28/132 (21.2)	27.3	(18.8, 39.1)
	90/135 (66.7)	28/132 (21.2)	45.5	(34.5, 54.8)
Maintenance of Clinical Remission per SF/APS	68/139 (48.9)	30/139 (21.6)	27.3	(18.0, 38.3)
	88/145 (60.7)	30/139 (21.6)	39.1	(27.9, 48.4)
Endoscopic Response	60/221 (27.3)	16/223 (7.2)	20.1	(14.1, 27.0)
	93/229 (40.7)	16/223 (7.2)	33.5	(27.1, 40.4)
Endoscopic Remission	41/221 (18.6)	12/223 (5.4)	13.2	(8.0, 19.5)
	70/229 (30.6)	12/223 (5.4)	25.2	(18.8, 31.6)
CDAI Remission and Endoscopic Remission	33/221 (15.0)	8/223 (3.6)	11.3	(6.8, 17.2)
	60/229 (26.2)	8/223 (3.6)	22.6	(16.6, 28.5)

Benefit at Week 52

	UPA QD LS Mean (N)	PBO LS Mean (N)	UPA QD vs PBO LS Mean Diff	UPA QD vs PBO 95% CI
IBDQ	54.8 (98)	43.2 (52)	11.6	(4.2, 19.0)
	61.6 (126)	43.2 (52)	18.4	(11.3, 25.6)
FACIT-F	12.4 (98)	10.2 (51)	2.2	(-0.4, 4.7)
	14.7 (126)	10.2 (51)	4.5	(2.0, 6.9)

Risk at Week 52

	UPA QD n/N (%)	PBO n/N (%)	UPA QD vs PBO Diff	UPA QD vs PBO 95% CI
Serious Infections	10/221 (4.5)	11/223 (4.9)	-0.4	(-4.40, 3.50)
	16/229 (7.0)	11/223 (4.9)	2.1	(-2.30, 6.40)
Herpes Zoster	9/221 (4.1)	5/223 (2.2)	1.8	(-1.40, 5.10)
	22/229 (9.6)	5/223 (2.2)	7.4	(3.10, 11.60)
Adjudicated GI Perforations	3/221 (1.4)	1/223 (0.4)	0.9	(-0.90, 2.70)
	1/229 (0.4)	1/223 (0.4)	0.0	(-9.30, 9.30)
Malignancies excluding NMSC	3/221 (1.4)	1/223 (0.4)	0.9	(-0.90, 2.70)
	3/229 (1.3)	1/223 (0.4)	0.9	(-0.90, 2.60)
NMSC	0/221	1/223 (0.4)	-0.4	(-9.90, 9.00)
	2/229 (0.9)	1/223 (0.4)	0.4	(-8.90, 9.70)
Adjudicated MACE	0/221	0/223	0.0	(0.00, 0.00)
	0/229	0/223	0.0	(0.00, 0.00)
Adjudicated VTE	0/221	0/223	0.0	(0.00, 0.00)
	1/229 (0.4)	0/223	0.4	(-8.80, 9.70)

APS, abdominal pain score; CD, Crohn's disease; CDAI, Crohn's Disease Activity Index; FACIT-F, Functional Assessment of Chronic Illness Therapy-Fatigue; IBDQ, Inflammatory Bowel Disease Questionnaire; LS, least squares; MACE, major adverse cardiovascular event; NMSC, nonmelanoma skin cancer; PBO, placebo; QD, once daily; SF, stool frequency; UPA, upadacitinib; VTE, venous thromboembolic event.

Figure S11. Benefit-risk during induction in the overall population of patients with ulcerative colitis.

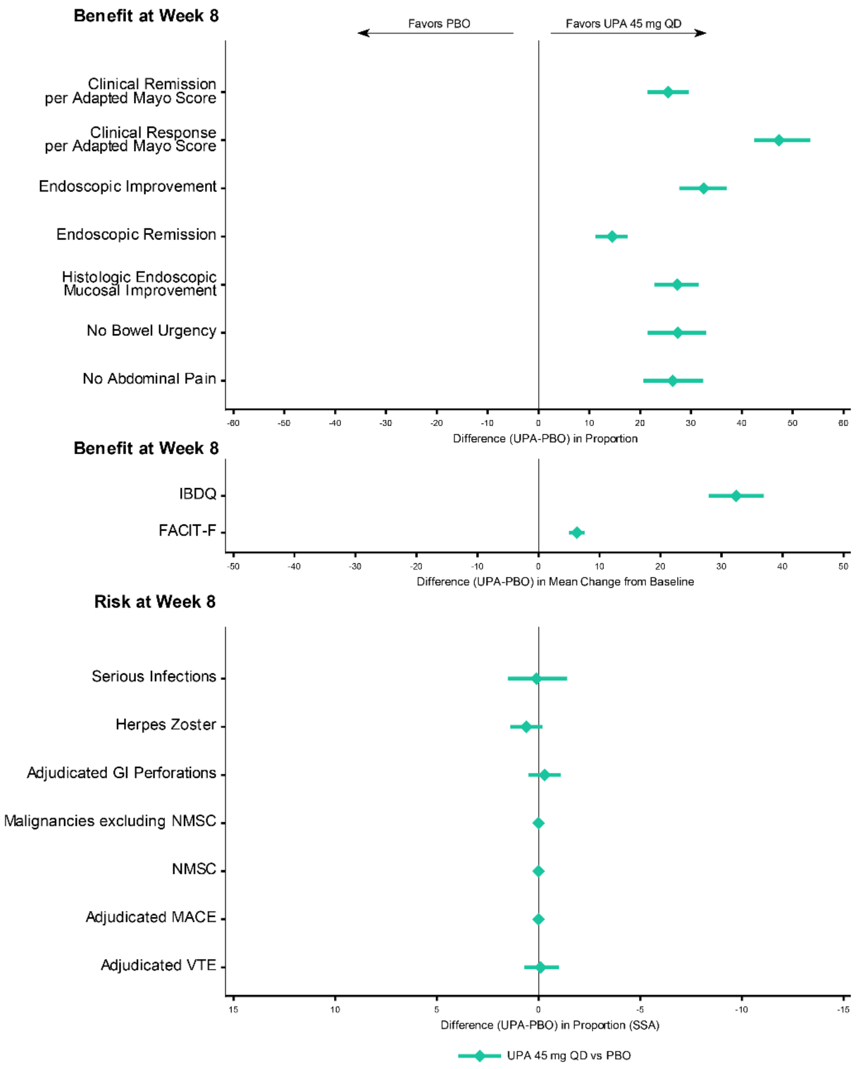

Benefit at Week 8

	UPA 45 mg QD n/N (%)	PBO n/N (%)	UPA 45 mg QD vs PBO Diff	95% CI
Clinical Remission per Adapted Mayo Score	198/660 (29.9)	15/328 (4.4)	25.5	(21.4, 29.6)
Clinical Response per Adapted Mayo Score	486/660 (73.6)	86/328 (26.2)	47.3	(42.4, 53.5)
Endoscopic Improvement	266/660 (40.3)	26/328 (7.9)	32.5	(27.7, 37.0)
Endoscopic Remission	106/660 (16.0)	5/328 (1.6)	14.5	(11.2, 17.5)
Histologic Endoscopic Mucosal Improvement	221/660 (33.5)	20/328 (6.2)	27.3	(22.8, 31.5)
No Bowel Urgency	338/660 (51.2)	78/328 (23.8)	27.4	(21.5, 33.0)
No Abdominal Pain	331/660 (50.2)	78/328 (23.8)	26.4	(20.6, 32.4)

Benefit at Week 8

	UPA 45 mg QD LS Mean (N)	PBO LS Mean (N)	UPA 45 mg QD vs PBO LS Mean Diff	95% CI
IBDQ	53.7 (607)	21.4 (281)	32.4	(27.8, 36.9)
FACIT-F	9.5 (603)	3.2 (280)	6.3	(5.0, 7.6)

Risk at Week 8

	UPA 45 mg QD n/N (SSA)	PBO n/N (SSA)	UPA 45 mg QD vs PBO Diff	95% CI
Serious Infections	9/719 (1.3)	5/378 (1.2)	0.1	(-1.40, 1.50)
Herpes Zoster	4/719 (0.6)	0/378	0.6	(-0.20, 1.40)
Adjudicated GI Perforations	0/719	1/378 (0.3)	-0.3	(-1.10, 0.50)
Malignancies excluding NMSC	0/719	0/378	0.0	(0.00, 0.00)
NMSC	0/719	0/378	0.0	(0.00, 0.00)
Adjudicated MACE	0/719	0/378	0.0	(0.00, 0.00)
Adjudicated VTE	1/719 (0.2)	1/378 (0.3)	-0.1	(-1.00, 0.70)

FACIT-F, Functional Assessment of Chronic Illness Therapy-Fatigue; IBDQ, Inflammatory Bowel Disease Questionnaire; LS, least squares; MACE, major adverse cardiovascular event; NMSC, nonmelanoma skin cancer; PBO, placebo; QD, once daily; SSA, study-size adjusted; UC, ulcerative colitis; UPA, upadacitinib; VTE, venous thromboembolic event.

Figure S12. Benefit-risk during maintenance in the overall population of patients with ulcerative colitis.

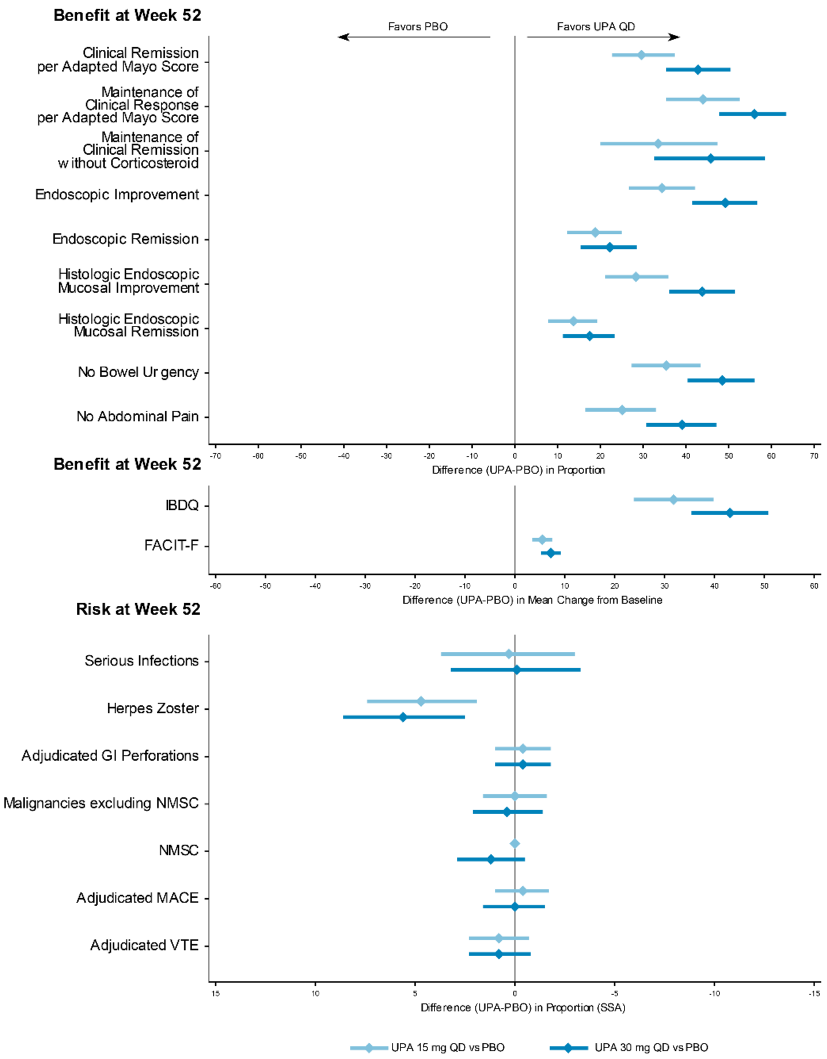

Benefit at Week 52

	UPA QD n/N (%)	PBO n/N (%)	UPA QD vs PBO Diff	UPA QD vs PBO 95% CI
Clinical Remission per Adapted Mayo Score	91/225 (40.4)	24/223 (10.8)	29.6	(22.7, 37.4)
	125/233 (53.6)	24/223 (10.8)	42.8	(35.4, 50.4)
Maintenance of Clinical Response per Adapted Mayo Score	129/197 (65.6)	44/204 (21.5)	44.0	(35.4, 52.5)
	168/217 (77.5)	44/204 (21.5)	56.0	(47.8, 63.4)
Maintenance of Clinical Remission without Corticosteroid	40/76 (52.3)	16/85 (18.8)	33.5	(20.0, 47.3)
	56/87 (64.6)	16/85 (18.8)	45.8	(32.6, 58.5)
Endoscopic Improvement	109/225 (48.5)	31/223 (14.1)	34.4	(26.7, 42.1)
	147/233 (63.3)	31/223 (14.1)	49.2	(41.4, 56.7)
Endoscopic Remission	56/225 (24.9)	14/223 (6.1)	18.8	(12.2, 25.0)
	66/233 (28.3)	14/223 (6.1)	22.2	(15.4, 28.5)
Histologic Endoscopic Mucosal Improvement	91/225 (40.5)	27/223 (12.3)	28.3	(21.1, 35.9)
	131/233 (56.0)	27/223 (12.3)	43.8	(36.1, 51.5)
Histologic Endoscopic Mucosal Remission	42/225 (18.8)	11/223 (5.1)	13.7	(7.8, 19.3)
	53/233 (22.6)	11/223 (5.1)	17.5	(11.2, 23.3)
No Bowel Urgency	121/225 (53.8)	41/223 (18.4)	35.4	(27.3, 43.4)
	156/233 (66.9)	41/223 (18.4)	48.5	(40.4, 56.0)
No Abdominal Pain	103/225 (45.8)	46/223 (20.6)	25.1	(16.5, 33.0)
	139/233 (59.7)	46/223 (20.6)	39.1	(30.7, 47.1)

Benefit at Week 52

	UPA QD LS Mean (N)	PBO LS Mean (N)	UPA QD vs PBO LS Mean Diff	UPA QD vs PBO 95% CI
IBDQ	48.2 (225)	16.3 (223)	31.8	(23.9, 39.8)
	59.4 (233)	16.3 (223)	43.1	(35.4, 50.8)
FACIT-F	8.1 (225)	2.6 (223)	5.5	(3.5, 7.5)
	9.8 (233)	2.6 (223)	7.2	(5.3, 9.2)

Risk at Week 52

	UPA QD n/N (SSA)	PBO n/N (SSA)	UPA QD vs PBO Diff	UPA QD vs PBO 95% CI
Serious Infections	9/250 (3.6)	8/245 (3.3)	0.3	(-3.00, 3.70)
	8/251 (3.2)	8/245 (3.3)	-0.1	(-3.30, 3.20)
Herpes Zoster	12/250 (4.7)	0/245	4.7	(1.90, 7.40)
	14/251 (5.6)	0/245	5.6	(2.50, 8.60)
Adjudicated GI Perforations	0/250	1/245 (0.4)	-0.4	(-1.80, 1.00)
	0/251	1/245 (0.4)	-0.4	(-1.80, 1.00)
Malignancies excluding NMSC	1/250 (0.4)	1/245 (0.4)	0.0	(-1.60, 1.60)
	2/251 (0.8)	1/245 (0.4)	0.4	(-1.40, 2.10)
NMSC	0/250	0/245	0.0	(0.00, 0.00)
	3/251 (1.2)	0/245	1.2	(-0.50, 2.90)
Adjudicated MACE	0/250	1/245 (0.4)	-0.4	(-1.70, 1.00)
	1/251 (0.4)	1/245 (0.4)	0.0	(-1.50, 1.60)
Adjudicated VTE	2/250 (0.8)	0/245	0.8	(-0.70, 2.30)
	2/251 (0.8)	0/245	0.8	(-0.80, 2.30)

FACIT-F, Functional Assessment of Chronic Illness Therapy-Fatigue; IBDQ, Inflammatory Bowel Disease Questionnaire; LS, least squares; MACE, major adverse cardiovascular event; NMSC, nonmelanoma skin cancer; PBO, placebo; QD, once daily; SSA, study-size adjusted; UC, ulcerative colitis; UPA, upadacitinib; VTE, venous thromboembolic event.
